# Massively parallel single-cell B-cell receptor sequencing enables rapid discovery of diverse antigen-reactive antibodies

**DOI:** 10.1038/s42003-019-0551-y

**Published:** 2019-08-09

**Authors:** Leonard D. Goldstein, Ying-Jiun J. Chen, Jia Wu, Subhra Chaudhuri, Yi-Chun Hsiao, Kellen Schneider, Kam Hon Hoi, Zhonghua Lin, Steve Guerrero, Bijay S. Jaiswal, Jeremy Stinson, Aju Antony, Kanika Bajaj Pahuja, Dhaya Seshasayee, Zora Modrusan, Isidro Hötzel, Somasekar Seshagiri

**Affiliations:** 1 Molecular Biology, Genentech, South San Francisco, CA 94080 USA; 2 Bioinformatics & Computational Biology, Genentech, South San Francisco, CA 94080 USA; 3 Antibody Engineering, Genentech, South San Francisco, CA 94080 USA; 4 Department of Molecular Biology, SciGenom Labs, Cochin, Kerala 682037 India; 5 Present Address: SciGenom Research Foundation, Bangalore, 560099 India

**Keywords:** B-cell receptor, Next-generation sequencing

## Abstract

Obtaining full-length antibody heavy- and light-chain variable regions from individual B cells at scale remains a challenging problem. Here we use high-throughput single-cell B-cell receptor sequencing (scBCR-seq) to obtain accurately paired full-length variable regions in a massively parallel fashion. We sequenced more than 250,000 B cells from rat, mouse and human repertoires to characterize their lineages and expansion. In addition, we immunized rats with chicken ovalbumin and profiled antigen-reactive B cells from lymph nodes of immunized animals. The scBCR-seq data recovered 81% (*n* = 56/69) of B-cell lineages identified from hybridomas generated from the same set of B cells subjected to scBCR-seq. Importantly, scBCR-seq identified an additional 710 candidate lineages not recovered as hybridomas. We synthesized, expressed and tested 93 clones from the identified lineages and found that 99% (*n* = 92/93) of the clones were antigen-reactive. Our results establish scBCR-seq as a powerful tool for antibody discovery.

## Abstract (web summary)

Leonard Goldstein et al. use high-throughput single-cell B-cell receptor sequencing on thousands of individual B cells from rat, mouse, and human repertoires. They obtained paired full-length heavy- and light-chain variable regions, and show that this approach is a powerful tool for antibody discovery.

## Introduction

Antibody diversity is an important feature of the adaptive immune system. B cells produce a diverse array of antibodies by rearranging variable, diversity, and joining immunoglobulin germline gene segments^1–3^. Somatic hypermutation (SHM) and class switching add to antibody diversity. A mature antibody consists of two identical heavy chains linked through disulphide bonds and two identical light chains each linked to one of the heavy chains, generating two identical antigen-binding sites formed by the first immunoglobulin domain of each chain pair^2^. The heavy and light chains are encoded in separate gene loci, and each B cell normally expresses a single functional heavy and light chain sequence.

Next-generation sequencing has been applied to understand the diversity of the variable regions of heavy (VH) and light chains (VL) that determine the antigen specificity of antibodies. Until recently, the majority of high-throughput sequencing approaches produced unpaired VH and VL repertoires, as generating paired information requires obtaining data at the individual cell level^4^. Recently, techniques that isolate individual cells in microwell plates or droplets of an emulsion, followed by physical linking of VH and VL regions through overlap extension RT-PCR, have demonstrated the potential for obtaining VH–VL pairing information in a high-throughput manner^5–7^. However, these techniques only infer full-length variable region sequences indirectly, and single-cell information is lost during library construction. High-throughput approaches that yield full-length variable regions for individual B cells at scale would enable routine application of large-scale immune repertoire sequencing to antibody discovery and detailed repertoire characterization.

Here we describe the application of high-throughput single-cell sequencing to obtain the VH and VL sequences for antibodies from individual human, rat, and mouse B cells. We developed a bioinformatics framework to analyze the sequence data and identify accurate VH and VL pairing. Further, we show the utility of the technique for antibody discovery by expressing and testing predicted antigen-reactive antibody sequences. We demonstrate the potential of direct sequencing of individual antigen-reactive B cells to rapidly generate a large and diverse panel of antigen-specific antibody variable regions and thus expand immune repertoire sampling and expedite antibody discovery processes.

## Results

### High-throughput single-cell B-cell receptor sequencing

**Fig. 1:**
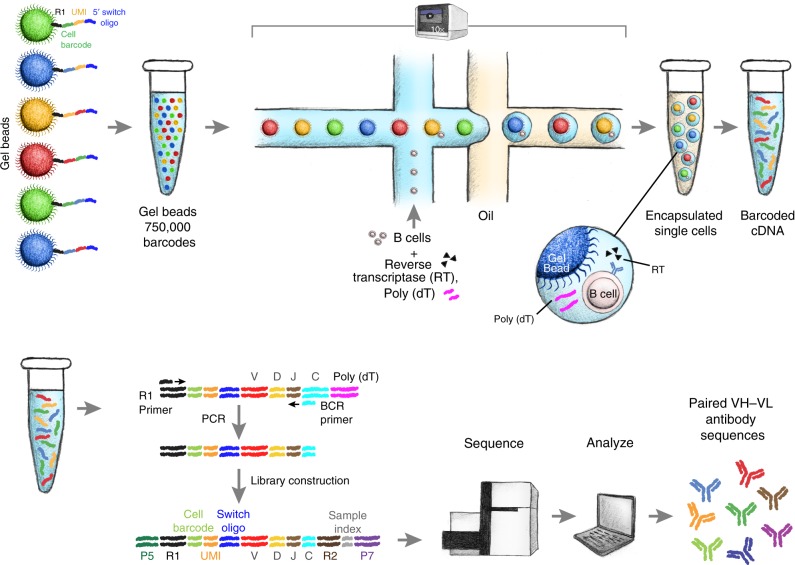
Schematic of single B cell capture, library construction and sequencing. Chromium controller (10x Genomics, Pleasanton, CA) was used to capture single cells along with gel beads containing the 5′ switch oligo with cell barcode, reverse transcriptase, and poly(dT) primer. The barcoded cDNAs were then converted into a library and sequenced (Illumina, San Diego, CA). Heavy- and light-chain sequences for individual cells were analyzed using a custom computational pipeline

**Fig. 2:**
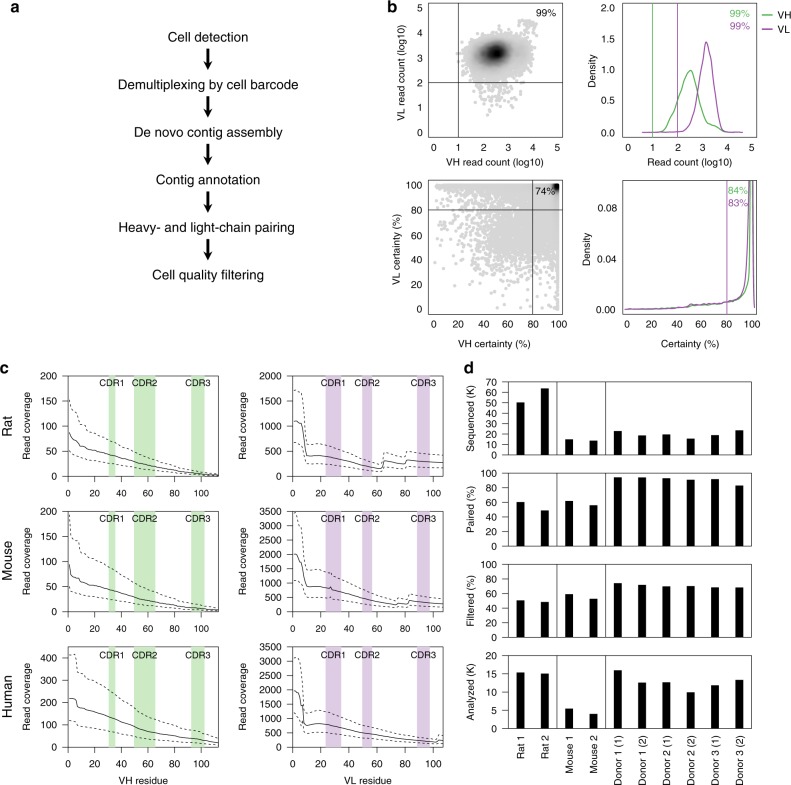
Computational framework for VH-VL pairing. **a** Data analysis workflow. **b** Cell quality filtering based on the number of reads for VH and VL assemblies and VH and VL certainty. Data shown are from one human sample, Donor 1 (1). **c** Read coverage for quality-filtered VH and VL assemblies for three different species. Positions within VH and VL are based on Kabat numbers, CDR regions are indicated by shaded regions. Solid lines indicate the median, dashed lines interquartile range. Data shown for rat, mouse, and human are from samples Rat 1, Mouse 1, and Donor 1 (1), respectively. **d** Overview of B-cell repertoire data generated for this study. Bar graphs show data for independent samples. From top to bottom, number of cells captured, percentage of cells with at least one complete VH and VL assembly, percentage of cells that pass quality filtering, number of cells that pass quality filtering

We analyzed >250,000 individual IgG^pos^ B cells from three human donors and two mice, and IgM^neg^ B cells from two rats using emulsion-based encapsulation, cDNA generation and sequencing. Briefly, we generated 5′ barcoded cDNA from thousands of individual B cells in parallel, and amplified the VH and VL regions using custom primers while retaining the cell barcode (Fig. 1, Supplementary Figs. 1, 2, and “Methods” section). The 5′ barcoded VH and VL domain-encoding cDNAs were sheared and converted into sequencing-ready libraries by addition of appropriate adapter oligonucleotides (Supplementary Fig. 1). The library construction method involves 3′ cDNA shearing after amplification to create a set of fragments with variable 3′ end, while retaining the 5′ end for all fragments. This resulted in sequencing reads with constant 5′ sequence and variable 3′ sequence, allowing de novo assembly of full-length VH and VL sequences from short-read data (2 × 150 bp). We devised a computational pipeline for cell detection, de novo contig assembly, variable domain annotation, and pairing of full-length VH and VL sequences (Fig. 2a, Supplementary Fig. 3). Assembled VH and VL sequences were parsed for framework regions and complementarity-determining regions (CDR) and selected for open reading frames encoding the entire variable region. Cells with complete VH and VL domains were filtered by requiring a minimum number of reads (10 and 100 for VH and VL, respectively) and requiring one dominant VH and VL contig (≥80% read support; Fig. 2b, Supplementary Fig. 4). As expected, filtered contigs had highest read coverage at the constant 5′ end and more variable coverage at the 3′ end (Fig. 2c, Supplementary Fig. 5). The median number of reads supporting filtered contigs was at least 175, 213, and 55 throughout the third CDR of the light chain (CDR-L3), and at least 3, 6, and 11 throughout the third CDR of the heavy chain (CDR-H3) for rat, mouse, and human B-cell repertoires, respectively (Fig. 2c, Supplementary Fig. 5). Pairing efficiency, defined as the percentage of cells with at least one detected VH and VL, was 48–94%, depending on sample type, and 48–74% of cells with pairing information passed quality filters (Fig. 2d). Variation in pairing and filtering efficiency may be due, in part, to differences in heavy- and light-chain transcript levels, primer efficiency, and primer coverage. Overall, a total of 261,539 sequenced B cells yielded high-quality VH–VL pairing information for 116,115 cells from rat (*n* = 30,380), mouse (*n* = 9459) and human (*n* = 76,276) B-cell repertoires (Fig. 2d, Supplementary Fig. 3, Supplementary Data 1–11).

### VH–VL pairing accuracy

**Fig. 3:**
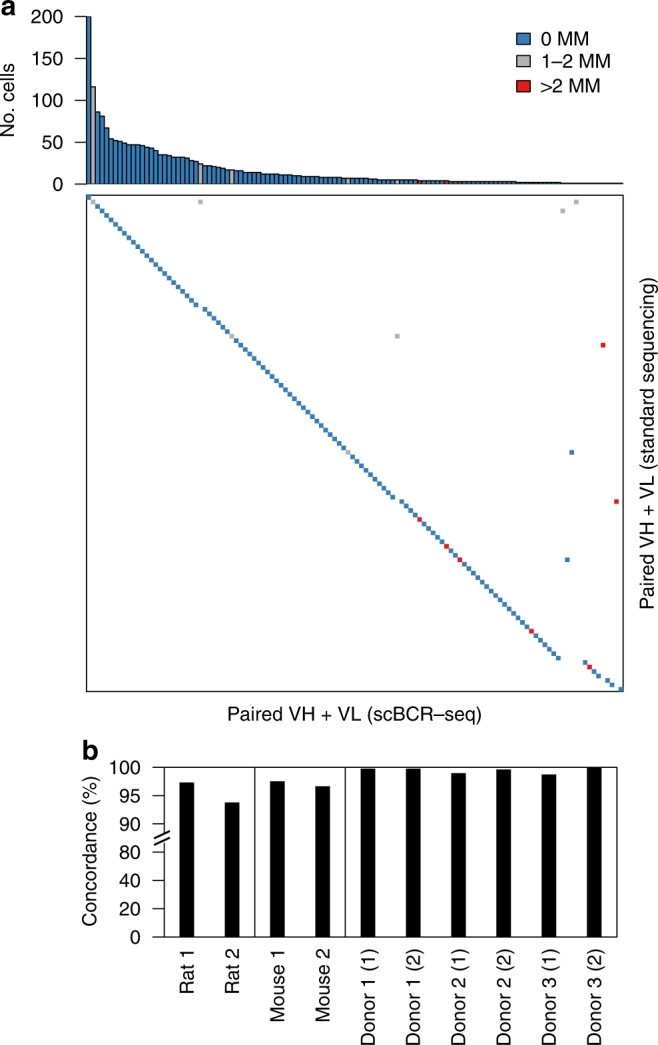
Technology validation. **a** Comparison of unique VH–VL nucleotide sequences obtained by scBCR-seq (columns) and reference VH–VL pairs obtained by a standard sequencing approach (rows). Blue, gray, and red boxes indicate VH–VL sequences validated with 0, 1–2, and >2 mismatches, respectively. Top panel shows number of cells for a particular VH–VL sequence in scBCR-seq data. **b** Pairing accuracy based on V_L_ concordance for VH–VL pairings with identical V_H_ and CDR-H3

To assess the accuracy of assembled VH and VL sequences and VH–VL pairing, we used hybridomas obtained after immunizing rats with chicken ovalbumin (OVA) as a test sample. We analyzed individual clones by a standard sequencing approach to obtain a reference set of paired VH–VL sequences for 127 unique clones (see “Methods”). The same clones were pooled, grown in culture, and subjected to scBCR-seq. The scBCR-seq approach yielded high-quality VH–VL sequence pairs for 1989 cells, representing 120 unique VH–VL sequence pairs. More than 90% of cells (1,801/1,989) showed a perfect match with one of the reference VH–VL pairs and more than 99% (1,972/1,989) matched one of the reference VH–VL pairs when allowing for up to two nucleotide mismatches (Fig. 3a).

Next, we assessed VH–VL pairing accuracy based on scBCR-seq data from human, mouse, and rat B-cell repertoires. Cells within the same B-cell lineage are expected to have light chains with concordant light-chain variable germline gene segment (V_L_) in the majority of cases^8^. We therefore assumed cells with identical heavy-chain variable germline gene segment (V_H_) and CDR-H3 belonged to clonally related B cells, and calculated the percentage of cells with concordant paired V_L_. Concordance in the subset of lineages with more than one B cell was >98% for human (based on 754–2131 cells in 351–894 lineages per sample), >96% for mouse (445 and 979 cells in 148 and 225 lineages, respectively, per sample), and >93% for rat (209 and 598 cells in 97 and 269 lineages, respectively, per sample) (Fig. 3b). These estimates are likely lower bounds for pairing accuracy, due to coincidental V_H_ and CDR-H3 matches in some B-cell lineages, in particular for short CDR-H3 sequences more prevalent in rodents^9^.

### Rat and mouse B-cell repertoires

**Fig. 4:**
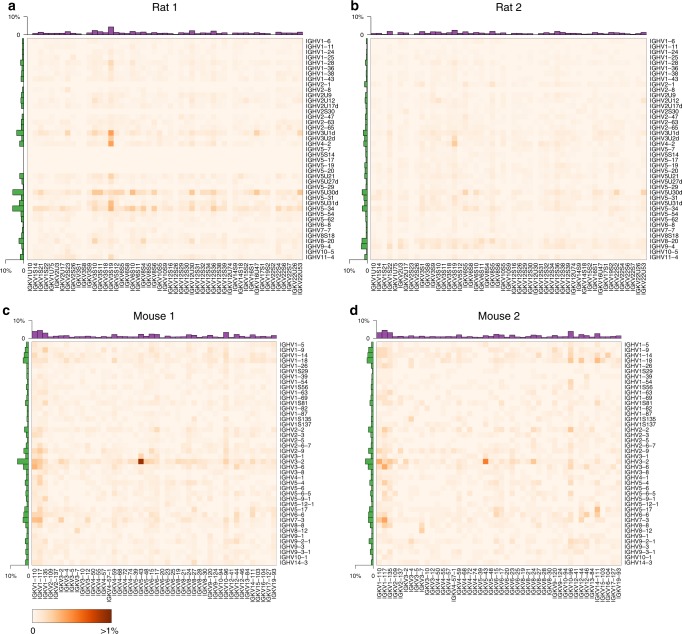
Variable germline gene segment pairing for B-cell repertoires from two rats (**a**, **b**) and two mice (**c**, **d**). Heatmaps show the percentage of lineages with a particular V_H_–V_L_ pairing. Row and column histograms indicate marginal V_H_ and V_L_ frequencies, respectively

We profiled IgM^neg^ B-cell repertoires from two nonimmunized rats (*n* = 30,380) and IgG^pos^ B-cell repertoires from two mice (*n* = 9459) (Fig. 2d, Supplementary Fig. 6a, b). Data at single-cell resolution allowed us to characterize unique B-cell lineages and quantify their expansion based on the number of cells observed for each lineage. We defined B-cell lineages by grouping cells with identical V_H_ and V_L_ germline gene segments and requiring at least 80% nucleotide identity in the CDR-H3 region. For the two rat samples, only 7% (1,081/15,338) and 3% (431/15,042) of cells belonged to clonally expanded lineages (Supplementary Fig. 7). For the two mouse samples, 13% (699/5,448) and 34% (1,353/4,011) of cells belonged to clonally expanded lineages, suggesting some level of antigenic stimulation in these mice (Supplementary Fig. 7). We asked whether the identified B-cell lineages showed preferential usage of particular V_H_ genes, V_L_ genes, or V_H_–V_L_ gene pairings. We observed that some germline gene segments were consistently used more frequently than others across replicates (Fig. 4). This was particularly noticeable for mouse V_H_ and V_L_ genes (*ρ* = 0.89, *ρ* = 0.84, Spearman correlation coefficient) and to a lesser extent for rat V_H_ genes (*ρ* = 0.52) (Supplementary Fig. 8). For example, *IGHV3-2* was the most commonly used mouse V_H_ gene in both animals, present in 6.2% and 4.4% of lineages, respectively (Fig. 4c, d). After correcting for variation in V_H_ and V_L_ gene usage, some individual V_H_–V_L_ gene pairings showed higher than expected frequencies across replicates (Supplementary Fig. 8). For example, both mouse samples showed increased frequencies for lineages with *IGHV8-12*:*IGKV3-7* (*n* = 9, *n* = 7) and *IGHV3-2*:*IGKV5-43* (*n* = 45, *n* = 16). Higher than expected pairings may be due to immune responses to common antigens. Consistent with this interpretation, *IGHV8-12*:*IGKV3-7* lineages showed evidence for expansion in both animals in 3/9 and 5/7 lineages, respectively.

### Human B-cell repertoires

**Fig. 5:**
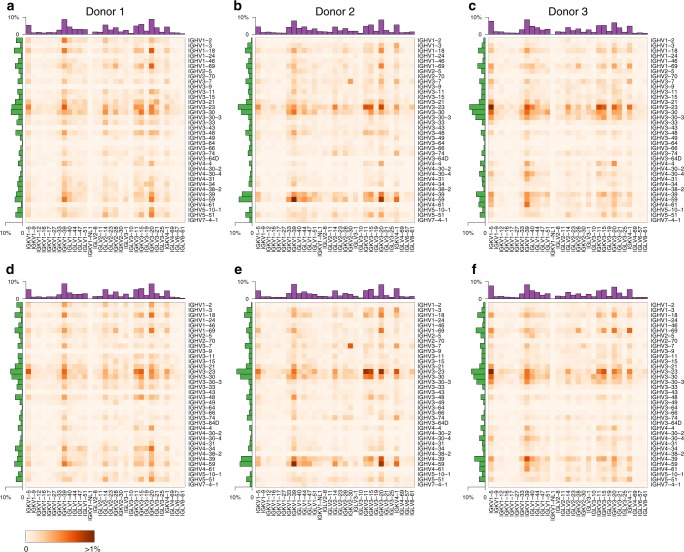
Variable germline gene segment pairing for B-cell repertoires from three human donors. Panels **a**–**c** and **d**–**f** show data from the same three donors at different time points, respectively. Otherwise as in Fig. 4

Next, we analyzed human IgG^pos^ B-cell repertoires from three donors, each profiled at two different time points (*n* = 76,276) (Fig. 2d, Supplementary Fig. 6c). All samples showed evidence for lineage expansion, with 14–26% of cells belonging to expanded lineages (Supplementary Fig. 7). V_H_ and V_L_ gene usage was highly reproducible between replicates from the same individual (*ρ* > 0.98, Supplementary Fig. 9). Overall V_H_ and V_L_ gene usage was similar among donors (*ρ* > 0.65 and *ρ* > 0.87 for V_H_ and V_L_, respectively, Supplementary Fig. 9) and in general agreement with the known distribution of germline usage in human repertoires^10,11^. Some donors lacked antibodies for a subset of germline gene segments (e.g., *IGHV3-9* in Donor 1 or *IGHV4-38-2* in Donor 2, Fig. 5), most likely due to genotype differences in the germline repertoires^11^. Interestingly all samples showed higher than expected pairing frequencies for *IGHV3-7*:*IGKV2-30* (19–62 lineages per sample) (Fig. 5, Supplementary Fig. 9). To rule out a technical artifact due to the profiling method, we reanalyzed published V_H_–V_L_ pairing information for naive and antigen-experienced human B cells that were obtained by overlap extension RT-PCR and independent computational methods^12^. Interestingly, the published data showed strongest enrichment for *IGHV3-7*:*IGKV2-30* among all variable germline gene segment pairings for antigen-experienced, but not naive B cells, suggesting this pairing may be the result of stereotypical immune responses (Supplementary Fig. 10).

### Rapid discovery of antigen-reactive antibody candidates

**Fig. 6:**
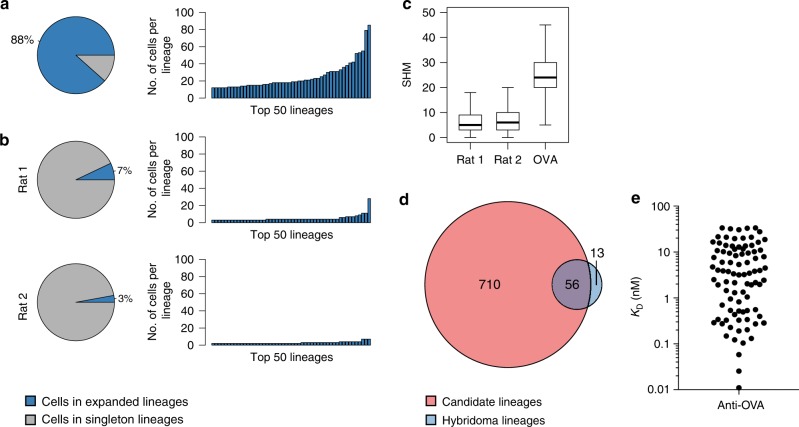
Discovery and validation of antigen-reactive antibodies. **a** Lineage expansions among OVA antigen-reactive B cells. Pie charts indicate percentage of cells belonging to expanded lineages. Bar charts indicate the number of cells for the top 50 lineages. **b** Lineage expansions observed in B-cell repertoires for two nonimmunized rats, otherwise as in **a**. **c** Somatic hypermutations (SHM) for heavy- and light-chain variable germline gene segments for B-cell repertoires from nonimmunized Rat 1 (*n* = 15,338) and Rat 2 (*n* = 15,042) and OVA antigen-reactive B cells pooled from three immunized rats (*n* = 3091). Boxes indicate the interquartile range (IQR), center lines the median, whiskers extend to the most extreme data point within 1.5 × IQR from the box. **d** Overlap in lineages identified from direct sequencing of individual antigen-reactive B cells by scBCR-seq (red) and concomitant hybridoma experiment (blue). **e** Validation of candidate OVA antigen-reactive clones. Shown are monovalent affinities of expressed antibodies to OVA

To assess the potential of scBCR-seq for antibody discovery, we immunized rats with chicken OVA and subjected IgM^neg^/OVA^pos^ lymph node B cells from three immunized animals to scBCR-seq (Supplementary Figs. 6d and 11). After quality filtering we obtained VH–VL pairing information for 3091 B cells (Supplementary Data 12). As expected, we observed substantial clonal expansion in this dataset with 88% of cells belonging to clonally expanded lineages (Fig. 6a). Of 766 unique B-cell lineages, 288 lineages (38%) were represented by three or more individual B cells (Supplementary Data 13). The top 73 lineages (10%) each included 10–85 B cells, comprising a total of 1494 cells. By contrast, IgM^neg^ B cells from naive rats showed limited evidence for clonal expansions (Fig. 6b). Chain pairing accuracy assessed by light-chain germline concordance was 99%, consistent with results obtained for naive rats. Somatic mutation load in the V_H_ and V_L_-derived regions (i.e., excluding regions containing CDR-H3, and the joining gene segments in both chains) was higher in anti-OVA cells than in IgM^neg^ B cells from naive rats (Fig. 6c). In addition to directly sequencing B cells from OVA-immunized animals, we also generated and sequenced OVA-specific hybridomas derived from a fraction of the IgM^neg^ B cells from the same rats. In this dataset we identified 69 unique B-cell lineages, 56 of which were shared with those identified by direct B-cell scBCR-seq (Fig. 6d, Supplementary Data 13). Thus scBCR-seq recovered 81% (56/69) of anti-OVA lineages from the hybridoma experiment, and identified an additional 710 candidate lineages.

### scBCR-seq identifies multiple antigen-reactive antibodies

We selected a set of 96 BCR (VH–VL) pairs identified from the OVA^pos^ B-cell scBCR-seq data for functional analysis. We selected the BCR sequence pairs from a range of lineage sizes observed in the scBCR-seq data (Supplementary Data 13). A random B-cell clone was selected from lineages represented by three or more individual B cells. For lineages with 1–2 cells, which may be enriched for antigen-negative clones due to imperfect cell sorting, we prioritized clones with high read count based on the assumption that these may be antibody-secreting cells induced by an active immune response. Of the 96 selected B cell receptor (BCR) sequence pairs, 77 were from lineages with three or more cells and 19 were from lineages with 1–2 cells. Of the 96 selected clones, 93 were from lineages not found in the hybridoma panel and 3 had nonidentical but clonally related OVA-specific monoclonal antibodies. Synthesized VH and VL DNA sequences were cloned into human IgG1/κ expression vectors and rat/human chimeric IgG was expressed in mammalian cells (see “Methods” section). We found that 93 of the 96 clones showed robust protein expression with a median yield of 85 µg of purified IgG per ml of culture (Supplementary Data 13). All but 4 of the 93 clones (96%) specifically bound OVA in an enzyme-linked immunosorbent assay (ELISA) (Supplementary Fig. 12a). To assess the importance of correct chain pairing for antigen binding in this panel, we shuffled each of the heavy and light chain expression constructs with a noncognate chain to generate 96 additional antibodies with nonnative light and heavy chain pairs. Eighty-six of the 96 shuffled clones expressed as IgG and only five of these showed OVA reactivity (Supplementary Fig. 12b). While some level of chain pairing promiscuity is expected^13^, the small number of shuffled clones with OVA reactivity confirms the relevance of accurate chain pairing for identification of antigen-binding antibodies. We further determined the affinity of the 93 expressed antibodies for OVA by surface plasmon resonance (SPR). Eighty-nine clones, including three clones with weak binding in ELISA, bound to OVA in SPR, with monovalent equilibrium dissociation constants (*K*_D_) ranging from the limit of detection (10 pM) to 30 nM (geometric mean *K*_D_, 2.4 nM; median *K*_D_, 3.5 nM) (Fig. 6e, Supplementary Fig. 12a, Supplementary Data 13). The observed affinities were similar to those of antibody panels with comparable size^14^. Interestingly, binding affinity did not correlate with read count, lineage size, or SHM load (Supplementary Fig. 13, Supplementary Data 13). However, as might be expected, B-cell clones with lower SHM loads (≤17 nt mutations in VH and VL combined) showed lower binding affinities (*K*_D_ ≥ 1.9 nM) in the tested panel (Supplementary Fig. 13d). Overall, 86 of the 93 expressed antibodies (92%) bound OVA in both ELISA and SPR, and 92 antibodies (99%) bound OVA in one of the two assays. These findings show scBCR-seq to be a robust tool for rapid discovery of a large panel of antigen-reactive antibodies.

## Discussion

Antibody discovery methods based on molecular cloning of antibody repertoires are limited by the ability to process large numbers of single cells in parallel. A number of next-generation sequencing approaches that capture paired antibody heavy- and light-chain information have been described. Techniques that employ manual sorting of cells into microwell plates, followed by deep single-cell RNA sequencing can yield high-quality full-length VH–VL sequence information, but are inherently low-throughput^15–17^. Recently, techniques that isolate single cells in microwell plates or droplets of an emulsion, followed by physical linking of VH and VL regions through overlap extension RT-PCR, have demonstrated the potential for obtaining VH–VL pairing information in a high-throughput manner^5–7^. However, such techniques require custom equipment and do not yield full-length variable region sequences or single-cell information. They allow chain pairing by paired-end sequencing of VH and VL sequences that are physically linked in a single amplicon. However, read length limitations of current sequencing technologies only allow partial sequencing of the variable regions. Full-length sequences can be inferred indirectly, through additional sequencing of VH and VL libraries constructed from the original VH–VL paired library, but with considerable uncertainty during sequence assembly^7^. Due to the lack of full-length variable region sequences for individual B cells, pairing information only applies to clonotypes, or groups of clonally related B-cell receptors, and information on clonal size and SHM load is partially lost. By contrast, scBCR-seq yields full-length variable region sequences at single-cell resolution. Key features of the approach are the partitioning of individual cells with barcoded reagents, and random fragmentation of VH and VL amplicons. Cell barcodes allow linking of heavy and light chain sequences and assignment to a single cell. Fragmentation of VH and VL amplicons generates 3′ end diversity, allowing assembly of full-length variable region sequences from short reads.

Low read coverage at the variable region 3′ end can result in partially truncated FR4 sequences, in particular for VH. However, unlike CDR regions that are directly involved in antigen binding, short missing FR4 regions can be reconstructed from germline sequences with minimal impact on antigen-binding properties. In future studies, it may be possible to improve read coverage through optimization of the experimental protocol. For example, reanalysis of a dataset generated by 10x Genomics, using a protocol with reduced fragmentation time, showed higher coverage in the third CDR region (Supplementary Fig. 14).

The scBCR-seq approach described here was performed using commercially available instruments and reagents (10x Genomics) with custom reagents limited to species-specific primers. Importantly, scBCR-seq does not require complex primer sets that cover the highly diverse V_H_ and V_L_ germline gene segments of human and rodent repertoires, minimizing biases and blind spots introduced in the amplification process. Thus scBCR-seq should be applicable with minimal modification to other species, including those for which only constant region sequences are known. Here we used custom primers for rat and mouse, and 10x Genomics primers for human. More recently, 10x Genomics released mouse primers, allowing a direct comparison between custom and commercial primers. Reanalysis of a mouse dataset generated by 10x Genomics (Supplementary Fig. 14) showed largely similar characteristics as mouse datasets generated with custom primers (Supplementary Figs. 3–5).

Our results demonstrate that scBCR-seq can be used for rapid discovery of large, diverse panels of high-affinity antigen-specific antibodies with natively paired heavy- and light-chains when combined with high-quality antigen-specific B-cell sorting. This is supported by the high rate of antigen binders and binding affinities and the high overlap between the anti-OVA B-cell repertoire and hybridoma sequencing. The highly consistent V_L_ germline pairing with clonally related heavy chains support the high quality of chain pairing information yielded.

Sequence panels of tens of thousands of individual B cells can be obtained by scBCR-seq within a week from cell harvest, with an additional 3 weeks for DNA synthesis, recombinant IgG expression and purification, and initial testing of binding properties. The yield of sequences from antigen-specific cells is limited by cell sorting capacity, which is impacted by the usually low frequency of antigen-positive B cells or plasmablasts enriched in antigen-specific clones in immunized animals and donors^18–21^. The frequency of antigen-specific cells is expected to be lower for cell types such as plasma cells, which cannot be antigen-sorted prior to sequencing.

Here we only tested a subset of the diversity yielded by scBCR-seq. Clones were selected for validation to assess the overall sequence quality in the panel, including pairing reliability. Within each unique lineage, clones were selected randomly. Still, 30% of the selected clones bound with monovalent *K*_D_ of 1 nM or better. Although not attempted here, sequence information, including lineage size, SHM load, and read counts per cell, as possible proxies for memory B cells and plasmablasts, or a combination of these, may be useful in selecting clones with higher affinities. In our dataset we did not find clear correlations between these factors and clone affinity. In the case of SHM, similar findings have been previously reported^22^. However, clones tested here were from different lineages, which may have differing ranges of affinities imposed by epitope chemistry and structural properties. Therefore, SHM load may not necessarily correlate with affinities across lineages. Establishing the predictive value of each of these factors for selection of higher affinity clones requires additional testing. However, even in the absence of rational methods for selecting the highest affinity clones, we expect that, once antibodies with the desired epitope specificity are identified, follow-up analyses of clones within expanded antigen-specific B-cell lineages in the scBCR-seq panels will allow easy and rapid empirical identification of higher affinity variants^23^. The availability of datasets with thousands of paired-chain, full-length variable region sequences from individual antigen-specific B cells will allow detailed large-scale characterization of the dynamics of immune responses, and the functional impact of germline usage, clonal expansion, and somatic mutation.

## Methods

### Isolation of IgG^pos^ B cells from human blood

Blood was obtained from healthy human donors before and on day 6 or 7 after vaccine immunization with the seasonal influenza Fluzone (year 2018). Samples were obtained after written informed consent was provided and ethical approval granted from the Western Institutional Review Board. Blood samples were diluted in phosphate-buffered saline (PBS) at a 1:1 (volume/volume) ratio and layered on top of a Ficoll-Paque-PLUS medium cushion (density 1.077 g/ml, GE Healthcare Life Sciences). Samples were centrifuged for 30 min at room temperature at 400 *g* with a soft stop. The interface layer containing peripheral blood mononuclear cells was collected and resuspended in FACS staining buffer (PBS, 0.5% BSA, and 2 mM EDTA) for marker staining and cell sorting. Single-cell suspensions at 5 × 10^7^ cells/ml were stained with a cocktail of fluorochrome conjugated human antibodies (BD Biosciences, San Jose, CA), anti-human IgG APC, anti-human CD20 PE Cy7, anti-human CD4 APC Cy7, Propidium iodide (PI) P4864 (Sigma-Aldrich, St. Louis, MO). IgG^pos^ B cells were enriched using PE-dump channel cocktail antibodies to exclude granulocyte, monocyte/macrophage, dendritic, NK, and CD8 cell populations. IgG^pos^ B cells (100,000 cells from each donor) were sorted on a FACSAria II cell sorter (BD Biosciences, San Jose, CA) and collected for scBCR-seq (Supplementary Fig. 15).

### Preparation of naive Balb/c mouse and rat lymph node B cells

All animals used in this study were housed and maintained at Genentech in accordance with American Association of Laboratory Animal Care guidelines. All experimental studies were conducted under protocols approved by the Institutional Animal Care and Use Committee of Genentech Lab Animal Research in an Association for Assessment and Accreditation of Laboratory Animal Care International-accredited facility in accordance with the Guide for the Care and Use of Laboratory Animals and applicable laws and regulations. Axillary brachial, mesenteric, inguinal, iliac, and popliteal LNs were collected from 8–10-week-old female naive Balb/c mice or 8–10-week-old female naive Sprague Dawley rats. Single-cell suspensions were prepared by crushing LNs through 70 µm polyester mesh sterile cell strainers (6 Netwell inserts plate, Corning). Murine B cells were enriched by negative selection of IgM^pos^ cells with a mouse B cell isolation kit (Miltenyi Biotec, #130-090-862, Bergisch Gladbach, Germany), resuspended in FACS staining buffer at 5 × 10^7^ cells/ml, stained with a cocktail of fluorochrome conjugated anti-mouse B220 APC (BD Biosciences, San Jose, CA) anti-mouse IgG FITC (Bethyl Laboratories, Montgomery, TX) for sorting of IgG^pos^ B cells (100,000 cells) on a FACSAria II cell sorter (Supplementary Fig. 16). Rat B cells were enriched using a cocktail of biotinylated anti-rat CD4 (Clone OX35), biotinylated anti-rat CD8a (Clone OX-8), biotinylated anti-rat 11b/c (Clone OX42), biotinylated anti-rat CD161 (Clone 10/78), and biotinylated anti-rat granulocyte marker (Clone HIS48) antibodies (BD Biosciences, San Jose, CA) followed by magnetic separation (Miltenyi Biotec, San Diego, CA) using streptavidin beads, stained with anti-rat IgM (Clone G53-238, BD Biosciences, San Jose, CA) conjugated to PE Cy7, CD45RA-APC Cy7, a dump cocktail of antibodies (BV510 anti rat granulocyte (Clone HIS48, BD Biosciences, San Jose, CA), BV510 anti rat 11b/c (Clone OX42, BD Biosciences, San Jose, CA), BV510 anti rat CD161a (Clone 10/78, BD Bioscience, San Jose, CA), and BV510 anti rat CD4 (Clone OX35, BD Bioscience, San Jose, CA)) and sorted for B cells (100,000 cells) on a FACSAria II cell sorter (Supplementary Fig. 17).

### Immunization of rats and antigen-specific cell sorting

Three Sprague Dawley rats (Charles River, Hollister, CA) were immunized with 100 µg OVA (Sigma-Aldrich, St. Louis, MO) in Complete Freund’s adjuvant (BD, Franklin Lakes, NJ) followed by biweekly boosts of 50 µg OVA in incomplete Freund’s adjuvant divided in three sites (intraperitoneally, subcutaneously at base of tail, at nape of neck and in both hocks). Multiple lymph nodes were harvested from each rat 2 days after the last immunization, pooled, and enriched for B cells as described above. Half of the enriched B cells were stained with anti-rat IgM (Clone G53-238, BD Biosciences, San Jose, CA) conjugated to PE Cy7, APC-labeled OVA conjugated to Alexa Fluor 647 (Thermo Fisher Scientific, Waltham, MA), CD8-per CPCy5.5, CD45RA-APC Cy7, a dump cocktail of antibodies (BV510 anti-rat granulocyte (Clone HIS48, BD Biosciences, San Jose, CA), BV510 anti-rat 11b/c (Clone OX42, BD Biosciences, San Jose, CA), BV510 anti-rat CD161a (Clone 10/78, BD Bioscience, San Jose, CA), and BV510 anti-rat CD4 (Clone OX35, BD Bioscience, San Jose, CA)) and sorted for dump channel^neg^/CD8^neg^/CD45RA^pos^/IgM^neg^/OVA^pos^ cells in a FACSAriaIII sorter (BD, Franklin Lakes, NJ) (Supplementary Fig. 18). A total of 32,845 such IgM^neg^/OVA^pos^ events were detected from the three rats and processed for scBCR-seq. The other half of the B cells were used for hybridoma generation. IgM-negative B cells were purified from lymphocytes using biotinylated anti-rat IgM (Clone G53-238, BD Biosciences, San Jose, CA) and magnetic separation (Miltenyi Biotec, San Diego, CA) using streptavidin beads followed by fusion with Sp2ab mouse myeloma cells (Abeome, Athens, GA) via electrofusion (Harvard Apparatus, Holliston, MA). Fused cells were incubated at 37 °C, 7% CO_2_, overnight in Clonacell-HY Medium C (StemCell Technologies, Vancouver, BC, Canada), before centrifugation and resuspension in Clonacell-HY Medium E (StemCell Technologies, Vancouver, BC, Canada) supplemented with HAT (Sigma-Aldrich, St. Louis, MO) and plating into 12-well plates and allowed to grow at 37 °C, 7% CO_2_. Four days after plating, hybridomas were stained with anti-rat IgG (goat polyclonal, Jackson ImmunoResearch, West Grove, PA) conjugated to Alexa 488 and APC-labeled OVA conjugated to Alexa Fluor 647 (Thermo Fisher Scientific, Waltham, MA) (5 µg/ml staining concentration) and sorted for IgG^pos^/OVA^pos^ cells in a FACSAriaIII sorter (BD, Franklin Lakes, NJ) (Supplementary Fig. 19). A total of 649 IgG^pos^/OVA^pos^ hybridoma cells were individually deposited into 96-well plates containing Medium E (StemCell Technologies, Vancouver, BC, Canada). Supernatants were screened by ELISA against OVA 7 days after sorting. A total of 379 clones producing antibodies binding to OVA in ELISA were pooled and submitted to scBCR-seq. ELISA-positive hybridoma clones were also individually sequenced as previously described, leading to the identification of 127 unique clones^24^.

### Single-cell library construction and sequencing

Sample processing for single B cell receptor (BCR) V(D)J clonotype was done using Chromium Single Cell 5′ Library and the Gel Bead Kit following the manufacturer’s user guide (10x Genomics, Pleasanton, CA, CG000086_SingleCellVDJReagentKitsUserGuide_RevB). After FACS sorting, cells were spun down, resuspended in 3% fetal bovine serum (Sigma-Aldrich, St. Louis, MO)/phosphate buffer solution (Thermo Fisher Scientific, Waltham, MA) and subjected to cell quality control using Vi-CELL XR cell counter (Beckman Coulter, Brea, CA). All of the processed B cells had cell viability >90%. After determining cell density, cells were injected into eight channels for rat samples, and four channels for mouse and human samples, aiming to achieve ~6000 cells per channel. Gel Beads-in-Emulsion (GEMs) were formed in channels of a chip in the 10x Chromium instrument, and then collected into an Eppendorf plate for GEM reverse transcription (GEM-RT) reaction. After GEM clean up, GEM-RT products were subjected to two rounds of 14 PCR cycles using custom primers for rat and mouse, and 10x human BCR primers (10x Genomics, Pleasanton, CA) for human, followed by SPRIselect (Beckman Coulter, Brea, CA) beads clean up. The 10x human BCR primers cover human heavy chain isotypes and both κ and λ light chains. Mouse and rat λ chains, which comprise a relatively minor fraction of the repertoire in rodents, were not covered by custom primers (Supplementary Information). Single-cell BCR V(D)J Libraries were prepared following the manufacturer’s user guide (10x Genomics, Pleasanton, CA, CG000086_SingleCellVDJReagentKitsUserGuide_RevB), and profiled using the Bioanalyzer High Sensitivity DNA kit (Agilent Technologies, Santa Clara, CA) and quantified with Kapa Library Quantification Kit (Kapa Biosystems, Wilmington, MA). Libraries were sequenced by paired-end sequencing (2 × 150 bp) on an Illumina HiSeq2500 or HiSeq4000 (Illumina, San Diego, CA). BCL data were converted to demultiplexed FASTQ files using Illumina bcl2fastq 2.20.

### Cell count estimate

For each library we tallied the number of reads for expected cell barcode sequences. Barcodes from cell-containing droplets were identified as those with read count exceeding a library-specific cutoff, defined as 0.1 times the read count for barcodes at rank 150, the 97.5th percentile for 6000 targeted cells.

### Contig assembly

We trimmed the first 39 bases for read 1 covering the 16 nt cell barcode, 10 nt unique molecular identifier (UMI) and 13 nt switch oligo. Barcode and UMI sequences were retained for each read. Reads were demultiplexed based on perfect matches to one of the expected barcode sequences. Subsequent processing was done independently for each barcode. If reads for a given barcode exceeded 100,000, they were downsampled to 100,000. Reads were used as input for de novo assembly with SSAKE (3.8.5)^25^ with options ‘-p 1 -c 1 -w 1 -e 1.5’ and expected mean insert size 600. We defined concordant read pairs as those with both reads fully embedded in the assembly and paired in the expected orientation. Contigs without concordant read pairs were discarded. Contigs were trimmed to regions supported by concordant read pairs. In cases with more than one contiguous supported region, the region with highest number of concordant read pairs was selected.

### Contig annotation

Sequences were annotated with a custom bioinformatics pipeline for variable domain analysis (https://github.com/Genentech/Absolve). Sequences were aligned using HMMER (http://www.hmmer.org/) to Hidden Markov Models that were trained on heavy and light germline amino acid sequences from the International Immunogenetics (IMGT) database^26^ in order to determine chain identity, framework and CDR boundaries and residue Kabat numbering^27^. Sequence germline assignments were determined by aligning to IMGT heavy- and light-chain variable and joining germline gene segment sequences^28^ using the SSW library^29^ and selecting the highest scoring germline. In order to assess the fidelity of germline classifications, Absolve was benchmarked with a set of 2000 simulated VH sequences, including random combinations of 252 variable, 44 diversity and 13 joining human germline gene segments and alleles and random trimming and addition of nucleotides. Each sequence was mutated up to 40 times, preferentially in CDR regions, to yield a set of 80,000 in-frame VH sequences with a variable number of mutations without stop codons. Processing of the simulated dataset with Absolve and IgBlast^30^ yielded comparable V_H_ and J_H_ germline call error rates (<0.5% in V_H_ at the highest mutational load). Reference germlines were from the IGMT database except for 52 additional rat germlines mined from *Rattus norvegicus* whole genome contigs from GenBank and germline sequences deduced from Sprague Dawley rat repertoire sequencing data (I. Hötzel, unpublished) (Supplementary Information).

### Heavy- and light-chain pairing

We considered contigs identified as either heavy- or light-chain variable domains with HMM score ≥30. Identified variable domains were considered complete if they contained all of FR1 and the first four positions of FR4. The latter requirement ensures that a minimum of four codons match germline sequences without frameshifts immediately following the third CDR in both chains, providing enough sequence information to determine CDR-H3 and CDR-L3 boundaries with high confidence. Amino acid sequences downstream of FR4 position 4 were ignored. For each cell we reported the complete heavy- and light-chain variable domain with highest number of concordant read pairs. For each reported heavy- and light-chain variable domain we calculated a certainty score, defined as the number of read pairs supporting the top contig divided by the total number of read pairs for all identified heavy- and light-chain contigs, respectively. Filtered high-quality pairings are those with (1) top heavy chain supported by ≥10 read pairs (2) top light chain supported by ≥100 read pairs, and (3) certainty ≥80% for both heavy and light chain.

### Lineage definition

Filtered cells were grouped into lineage clusters. Two cells were grouped together if they had identical germline V_H_ and V_L_ genes, ignoring allele number, identical CDR-H3 length and ≥80% identical CDR-H3 sequence. J_H_ and J_L_ genes were ignored for lineage definition due to the lower sequence coverage in this region.

### Antibody expression and binding assays

Selected B-cell clones from the OVA immunization dataset were produced by DNA synthesis and cloned in mammalian expression vectors^31^ as chimeric human IgG1/kappa antibodies. Sequences with clearly incorrect FR4 terminal sequences were corrected based on the assigned joining germline gene segment information for that clone but no additional coding changes were introduced in sequences. Antibodies were transfected into Expi293F^TM^ cells (Thermo Fisher Scientific, Cat. A14635) at 1 ml scale and purified in batch mode by protein A chromatography^32,33^. Antibodies with mispaired chains were made by combining each of the 96 heavy chain clones with a light chain clone from a different anti-OVA antibody. Purified IgGs were tested for binding to OVA in ELISA and in an SPR assay using a BIAcore T200 apparatus (GE Life Sciences, Piscataway, NJ) in a protein A capture format. Purified IgG was immobilized by binding to protein A on chips and soluble monomeric OVA was used as analyte in SPR, using the single cycle kinetics method at 25 °C.

### Statistics and reproducibility

Replicates refer to independent biological samples. Rat and mouse repertoire datasets each include two samples from two distinct animals. The human repertoire dataset includes samples from three different donors, with two samples obtained at different time points for each donor, six samples in total. The antigen-positive dataset includes one sample pooled from three animals. Reproducibility was verified using replicate samples and publicly available datasets, as indicated. The number of single-cell libraries generated for each sample are listed in Supplementary Data 1.

### Reporting summary

Further information on research design is available in the Nature Research Reporting Summary linked to this article.

## Supplementary information

Supplementary Information

Description of additional supplementary items

Supplementary Data

Reporting Summary

## Data Availability

Rat and mouse datasets generated during the current study are available at the NCBI Sequence Read Archive under project number PRJNA544118. Human datasets generated during the current study have been deposited at the European Genome-phenome Archive under accession code EGAS00001003663. Source data underlying Figs. 2–6 are included in Supplementary Data 1–15.

## Code availability

The custom bioinformatics pipeline for variable domain analysis is available under the MIT License on GitHub (https://github.com/Genentech/Absolve).

## References

[1] Schroeder HW (2006). Similarity and divergence in the development and expression of the mouse and human antibody repertoires. Dev. Comp. Immunol..

[2] Schroeder HW, Cavacini L (2010). Structure and function of immunoglobulins. J. Allergy Clin. Immunol..

[3] Watson CT, Breden F (2012). The immunoglobulin heavy chain locus: genetic variation, missing data, and implications for human disease. Genes Immun..

[4] Reddy ST (2010). Monoclonal antibodies isolated without screening by analyzing the variable-gene repertoire of plasma cells. Nat. Biotechnol..

[5] DeKosky BJ (2013). High-throughput sequencing of the paired human immunoglobulin heavy and light chain repertoire. Nat. Biotechnol..

[6] DeKosky BJ (2015). In-depth determination and analysis of the human paired heavy- and light-chain antibody repertoire. Nat. Med.

[7] McDaniel JR, DeKosky BJ, Tanno H, Ellington AD, Georgiou G (2016). Ultra-high-throughput sequencing of the immune receptor repertoire from millions of lymphocytes. Nat. Protoc..

[8] Hershberg U, Luning Prak ET (2015). The analysis of clonal expansions in normal and autoimmune B cell repertoires. Philos Trans. R. Soc. Lond. B Biol. Sci..

[9] Zemlin M (2003). Expressed murine and human CDR-H3 intervals of equal length exhibit distinct repertoires that differ in their amino acid composition and predicted range of structures. J. Mol. Biol..

[10] Glanville J (2009). Precise determination of the diversity of a combinatorial antibody library gives insight into the human immunoglobulin repertoire. Proc. Natl Acad. Sci. USA.

[11] Boyd SD (2010). Individual variation in the germline Ig gene repertoire inferred from variable region gene rearrangements. J. Immunol..

[12] DeKosky BJ (2016). Large-scale sequence and structural comparisons of human naive and antigen-experienced antibody repertoires. Proc. Natl Acad. Sci. USA.

[13] de Kruif J (2009). Human immunoglobulin repertoires against tetanus toxoid contain a large and diverse fraction of high-affinity promiscuous V(H) genes. J. Mol. Biol..

[14] Scally SW (2017). Molecular definition of multiple sites of antibody inhibition of malaria transmission-blocking vaccine antigen Pfs25. Nat. Commun..

[15] Lindeman I (2018). BraCeR: B-cell-receptor reconstruction and clonality inference from single-cell RNA-seq. Nat. Methods.

[16] Neu KE (2019). Spec-seq unveils transcriptional subpopulations of antibody-secreting cells following influenza vaccination. J. Clin. Investig.

[17] Upadhyay AA (2018). BALDR: a computational pipeline for paired heavy and light chain immunoglobulin reconstruction in single-cell RNA-seq data. Genome Med.

[18] Greenstein JL, Leary J, Horan P, Kappler JW, Marrack P (1980). Flow sorting of antigen-binding B cell subsets. J. Immunol..

[19] Doucett VP (2005). Enumeration and characterization of virus-specific B cells by multicolor flow cytometry. J. Immunol. Methods.

[20] Amanna IJ, Slifka MK (2006). Quantitation of rare memory B cell populations by two independent and complementary approaches. J. Immunol. Methods.

[21] Wang B (2018). Functional interrogation and mining of natively paired human VH:VL antibody repertoires. Nat. Biotechnol..

[22] Poulsen TR, Meijer PJ, Jensen A, Nielsen LS, Andersen PS (2007). Kinetic, affinity, and diversity limits of human polyclonal antibody responses against tetanus toxoid. J. Immunol..

[23] Hsiao YC (2019). Immune repertoire mining for rapid affinity optimization of mouse monoclonal antibodies. MAbs.

[24] Chen Y (2018). Barcoded sequencing workflow for high throughput digitization of hybridoma antibody variable domain sequences. J. Immunol. Methods.

[25] Warren RL, Sutton GG, Jones SJ, Holt RA (2007). Assembling millions of short DNA sequences using SSAKE. Bioinformatics.

[26] Lefranc MP (2002). IMGT, the international ImMunoGeneTics database: a high-quality information system for comparative immunogenetics and immunology. Dev. Comp. Immunol..

[27] Kabat, E. A., Wu, T. T., Perry, H. M., Gottesman, K. S. & Foeller, C. in NIH Publication No. 91-3243 (1991).

[28] Lefranc MP, Ehrenmann F, Ginestoux C, Giudicelli V, Duroux P (2012). Use of IMGT((R)) databases and tools for antibody engineering and humanization. Methods Mol. Biol..

[29] Zhao M, Lee WP, Garrison EP, Marth GT (2013). SSW library: an SIMD Smith-Waterman C/C++ library for use in genomic applications. PLoS One.

[30] Ye J, Ma N, Madden TL, Ostell JM (2013). IgBLAST: an immunoglobulin variable domain sequence analysis tool. Nucleic Acids Res.

[31] Eaton DL (1986). Construction and characterization of an active factor VIII variant lacking the central one-third of the molecule. Biochemistry.

[32] Luan P (2018). Automated high throughput microscale antibody purification workflows for accelerating antibody discovery. MAbs.

[33] Bos AB (2015). Optimization and automation of an end-to-end high throughput microscale transient protein production process. Biotechnol. Bioeng..

