# Supplementary material for: Massively parallel single-cell B-cell receptor sequencing enables rapid discovery of diverse antigen-reactive antibodies
Source: Commun Biol. 2019 Aug 9;2:304. doi: 10.1038/s42003-019-0551-y (PMC6689056; doi:10.1038/s42003-019-0551-y)
Supplement: Supplementary file 1 — Supplementary Information [file 42003_2019_551_MOESM1_ESM.pdf]

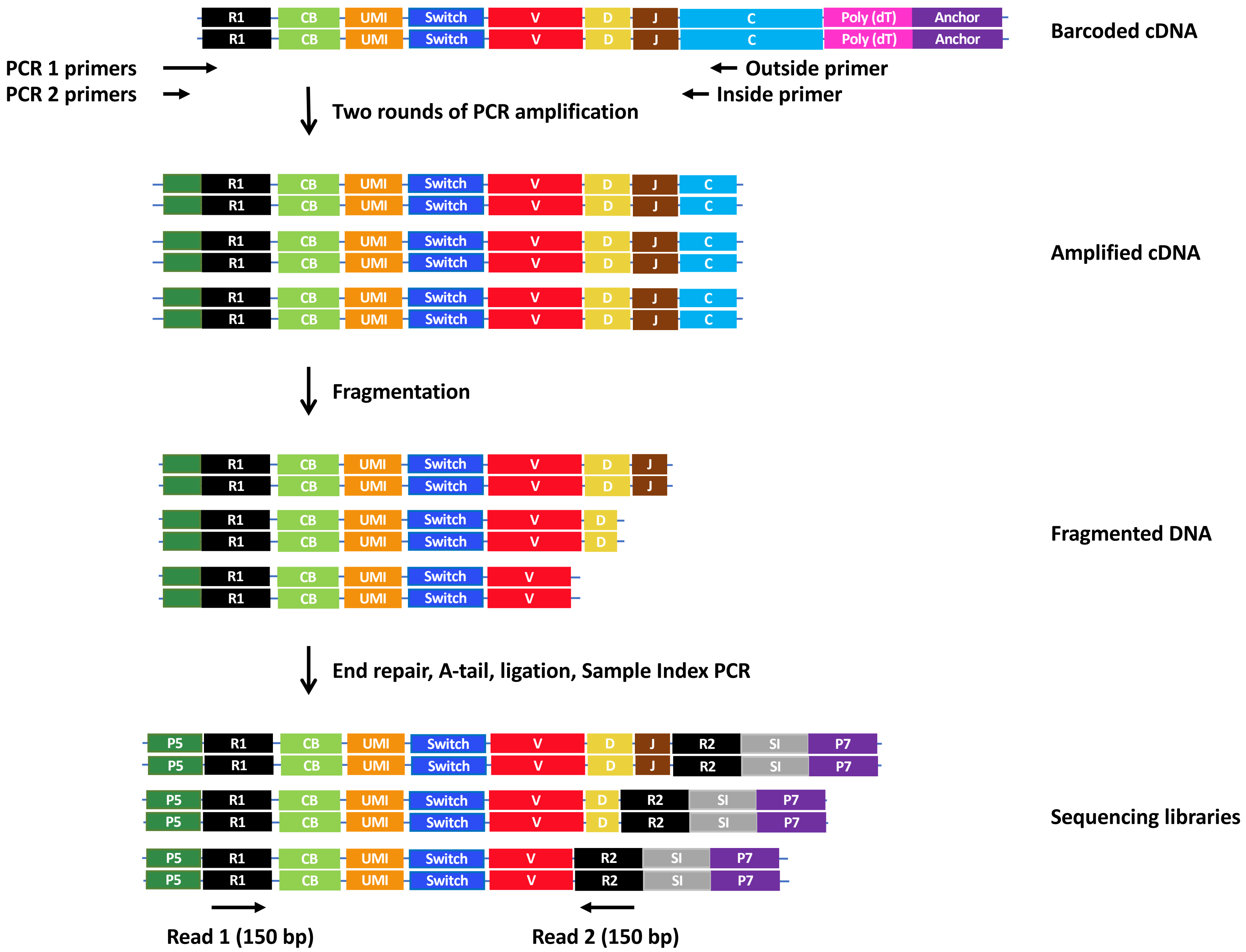

Supplementary Figure 1. Schematic of single-cell cDNA generation and library construction following the manufacturer's user guide (10x Genomics, Pleasanton, CA, CG000086_SingleCellVDJReagentKitsUserGuide_RevB). Only heavy chain is shown.

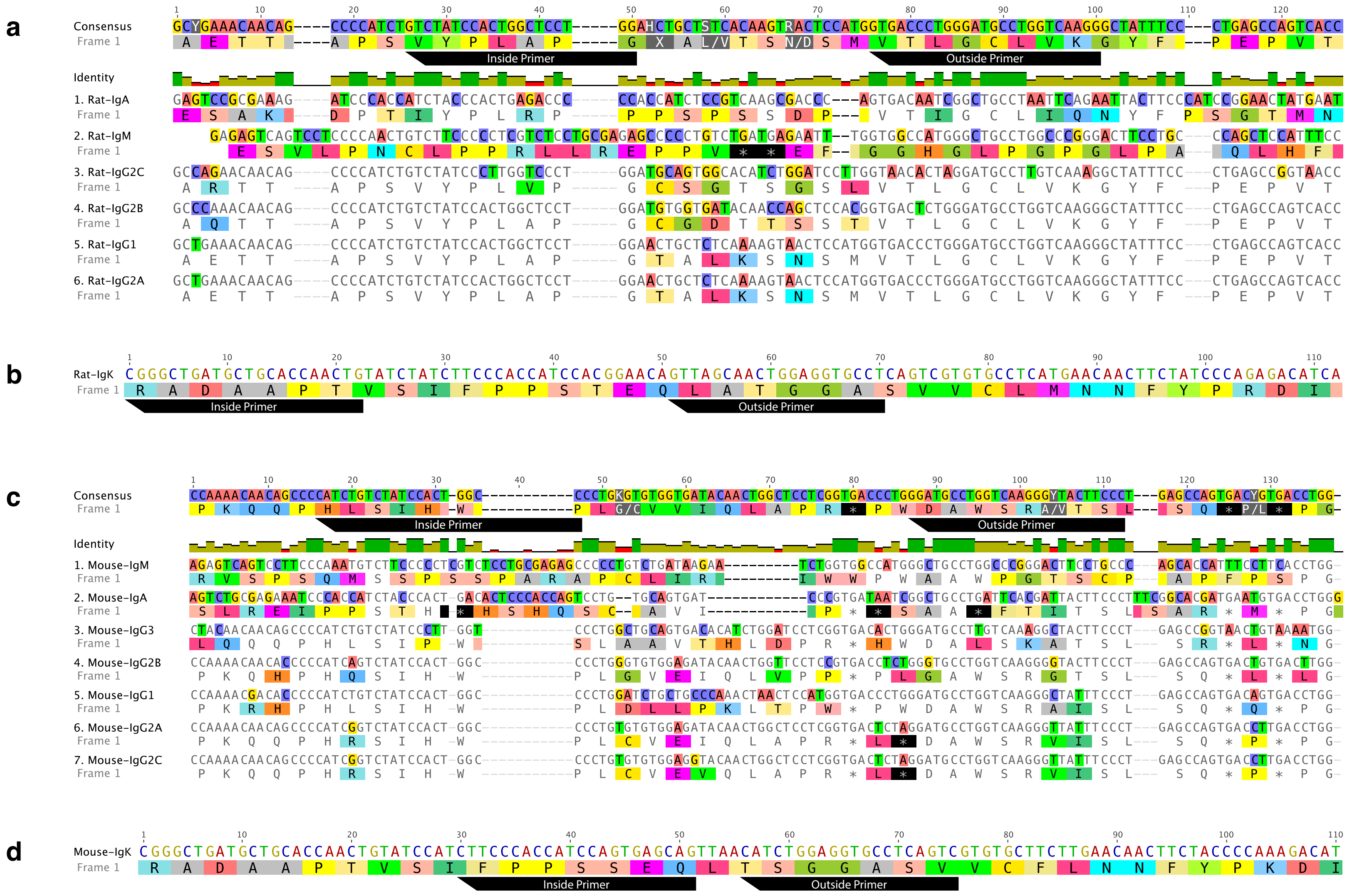

Supplementary Figure 2. Custom PCR primers targeting the rat and mouse heavy- and light-chain constant regions. (a) Multiple sequence alignment of rat heavy-chain isotype constant regions. (b) Rat κ light-chain constant region. (c) Multiple sequence alignment of mouse heavy-chain isotype constant regions. (d) Mouse κ light-chain constant region.

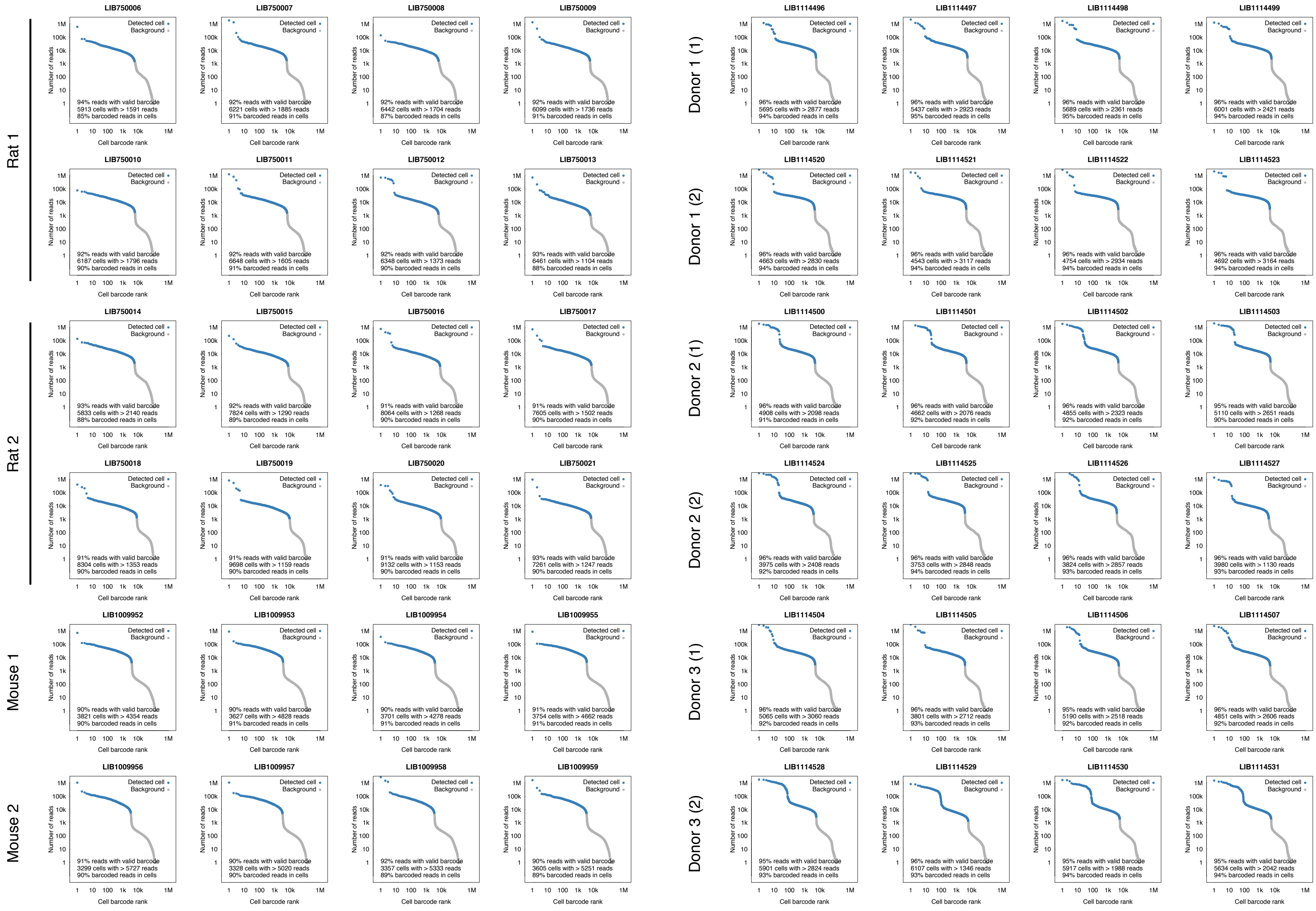

Supplementary Figure 3. Cell detection. Number of reads per barcode plotted against barcode rank for each library, barcodes for detected cells are colored in blue.

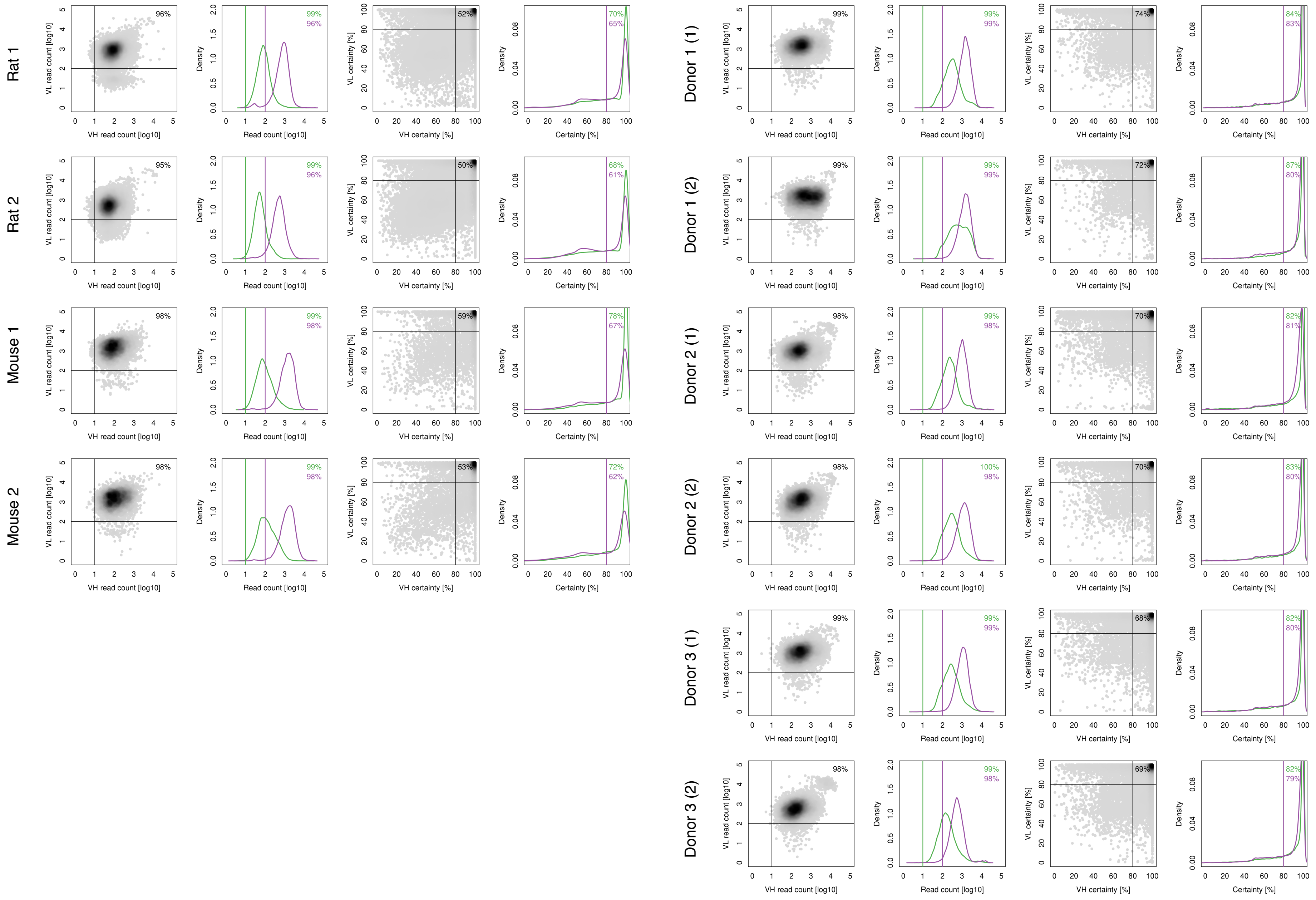

Supplementary Figure 4. Cell quality filtering. Otherwise as in Figure 2b.

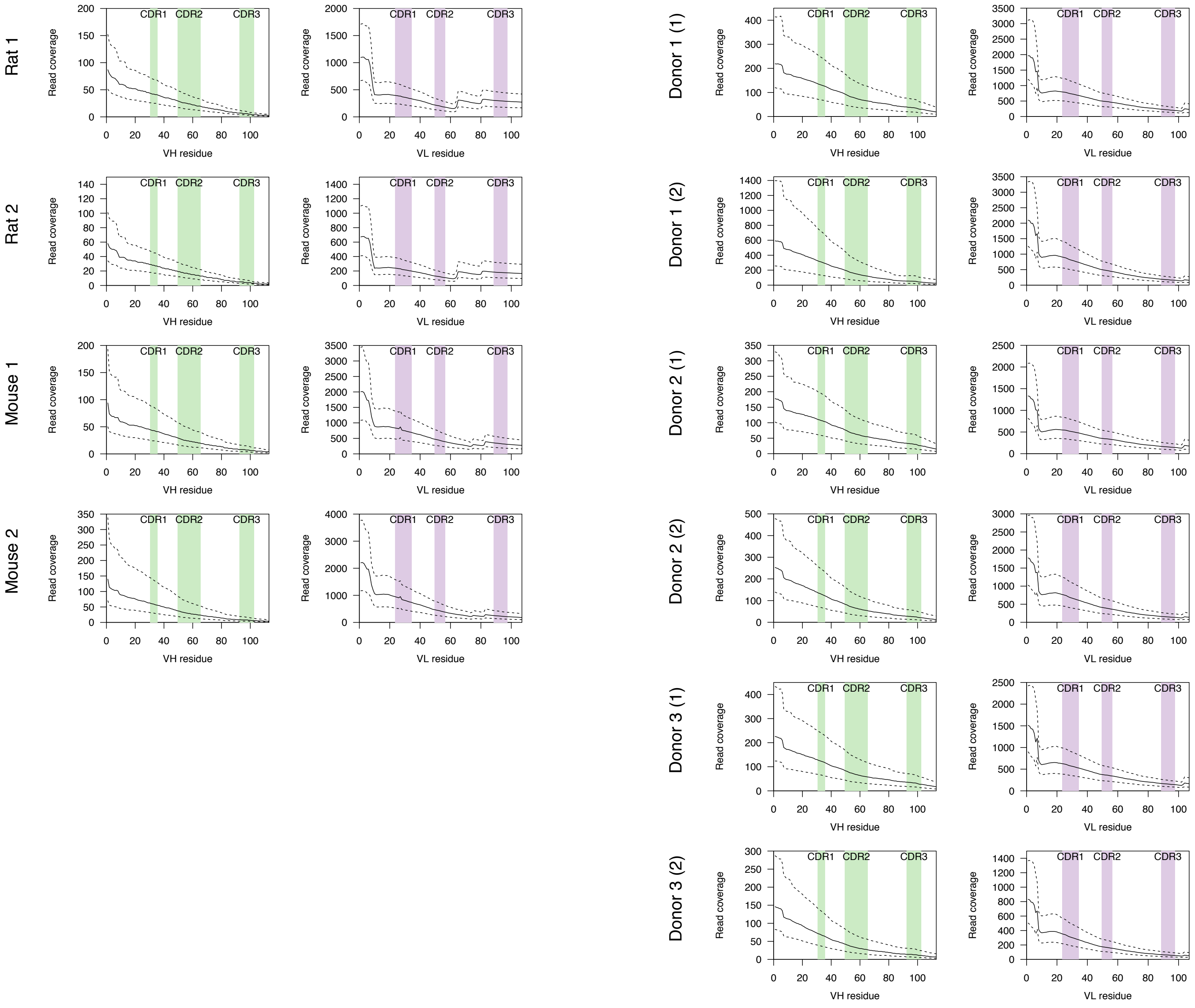

Supplementary Figure 5. Read coverage for quality-filtered VH and VL assemblies. Otherwise as in Figure 2c.

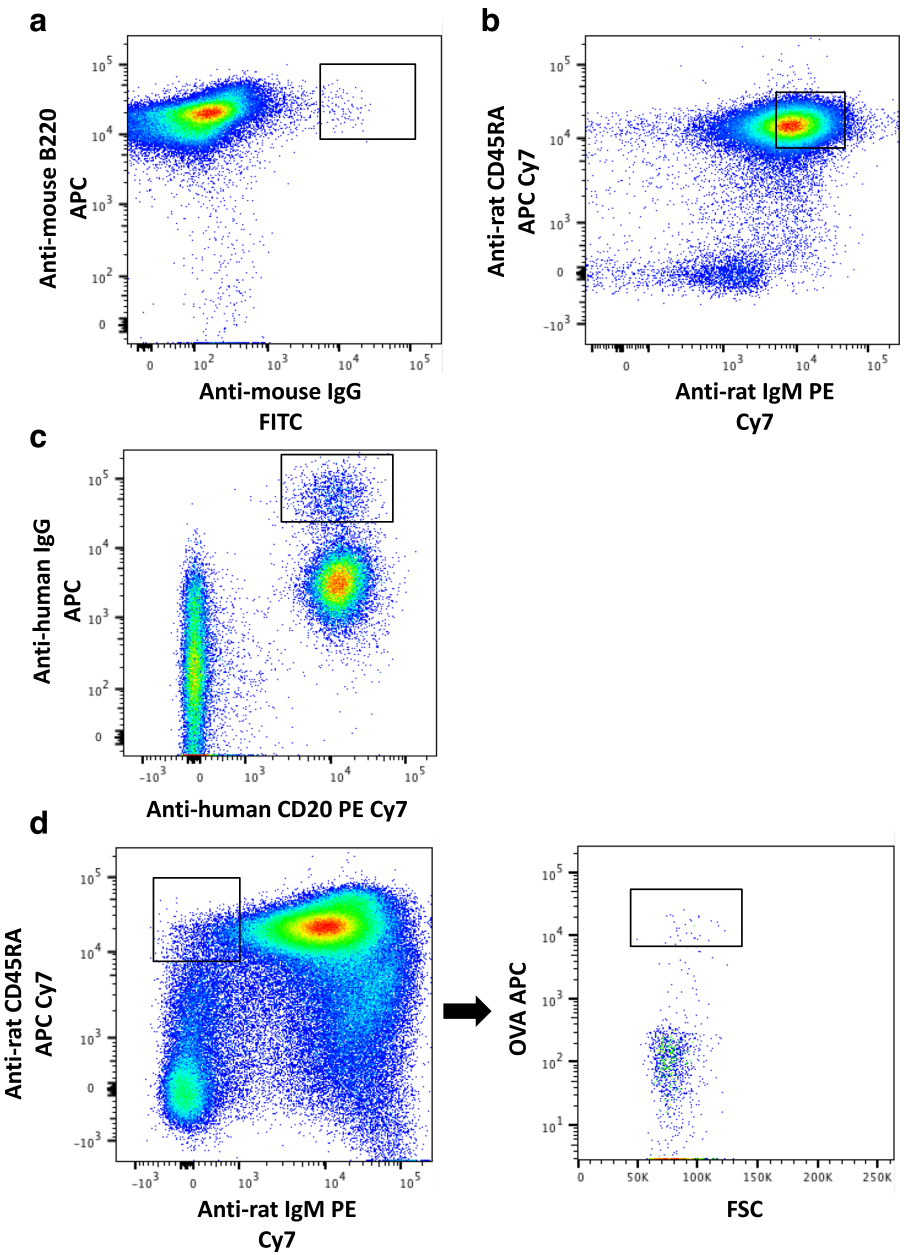

Supplementary Figure 6. Representative B cell sorting gates for naïve mice (a), naïve rat (b), human (c) and pooled OVA-immunized rats (d).

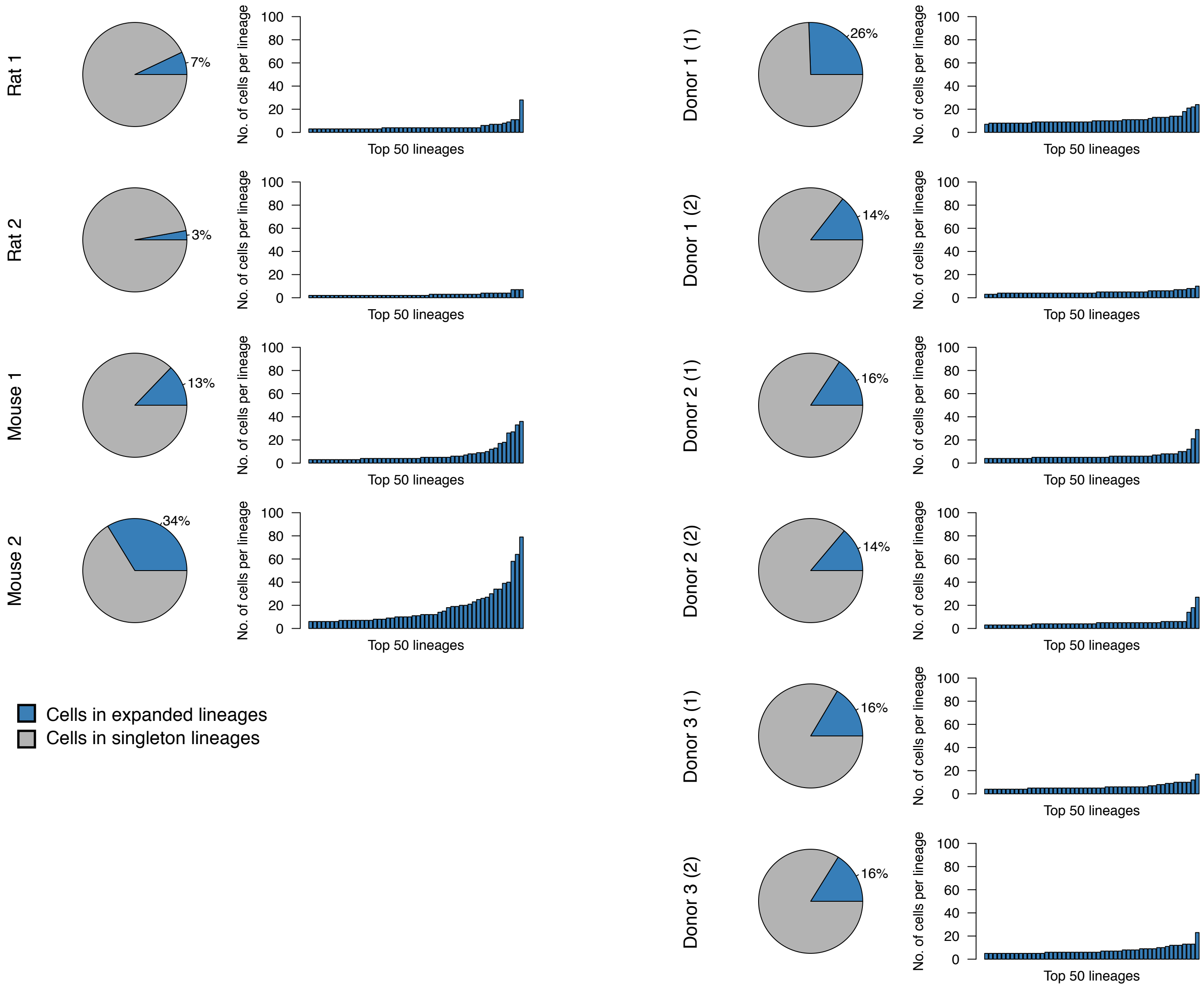

Supplementary Figure 7. Lineage expansions. Otherwise as in Figure 6a,b.

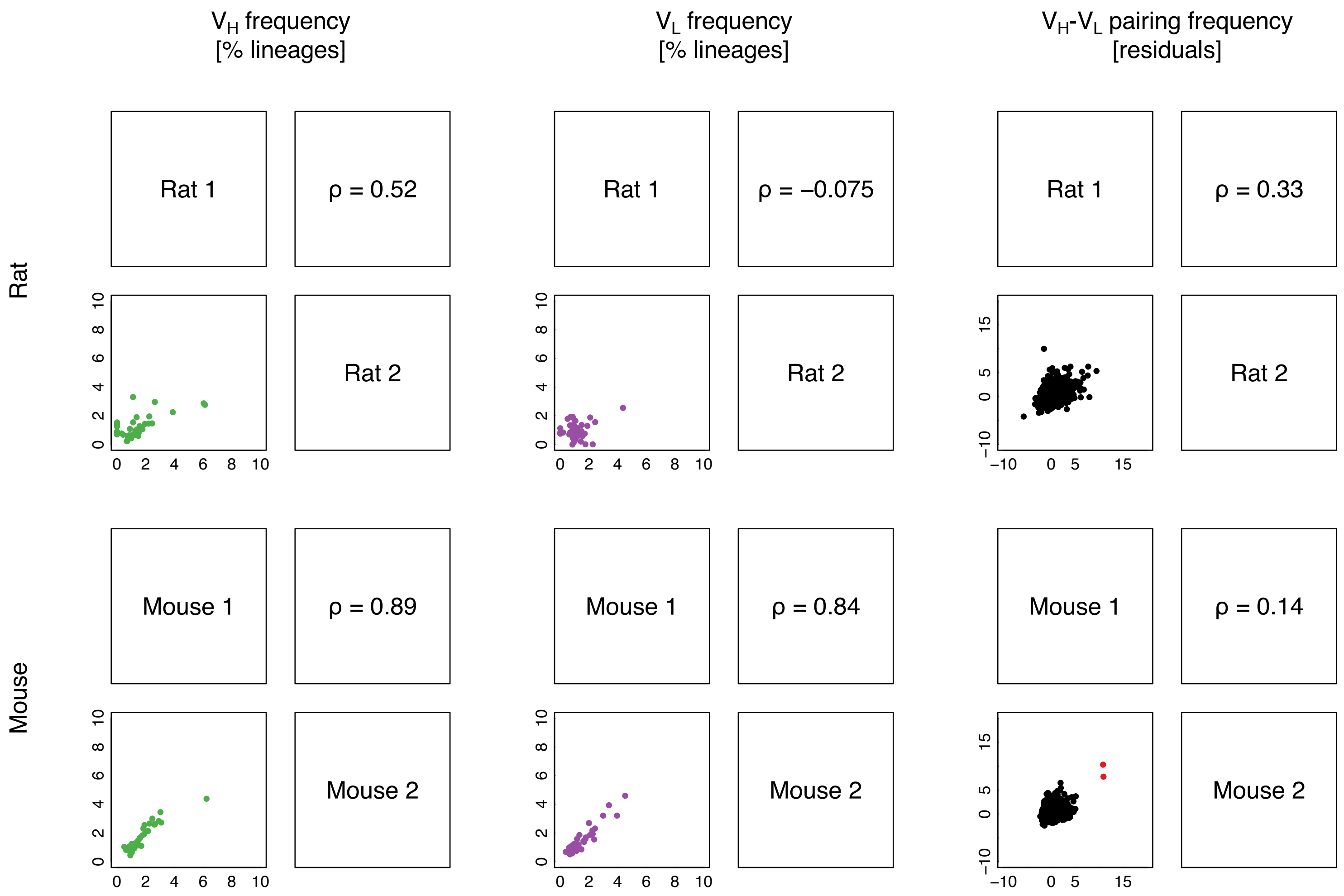

Supplementary Figure 8. Pairwise scatter plots for V_H frequencies, V_L frequencies, and standardized residuals for V_H-V_L pairing frequencies for two rats (top) and two mice (bottom). Frequencies are for lineages with a particular germline gene segment or pairing. *IGHV8-12:IGKV3-7* and *IGHV3-2:IGKV5-43* are highlighted in red.

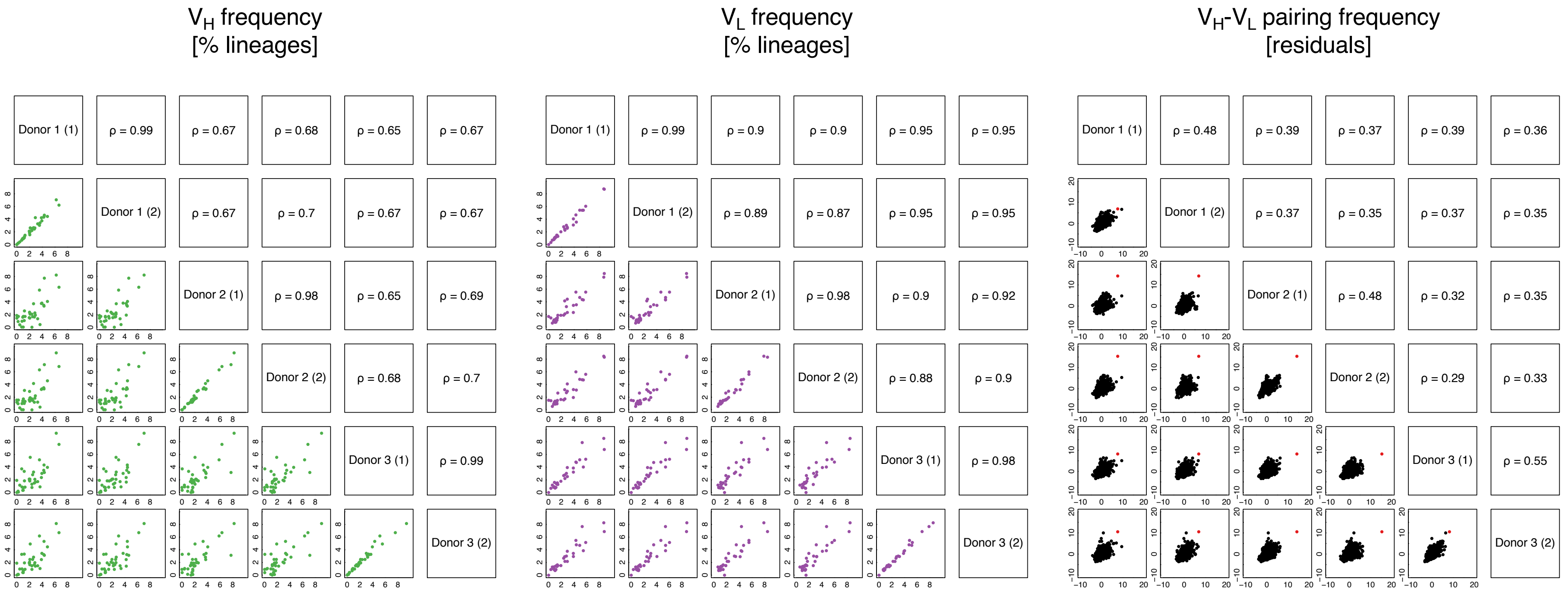

Supplementary Figure 9. Pairwise scatter plots for V_H frequencies, V_L frequencies, and standardized residuals for V_H-V_L pairing frequencies for three human donors, each profiled at two different time points. Frequencies are for lineages with a particular germline gene segment or pairing. *IGHV3-7:IGKV2-30* is highlighted in red.

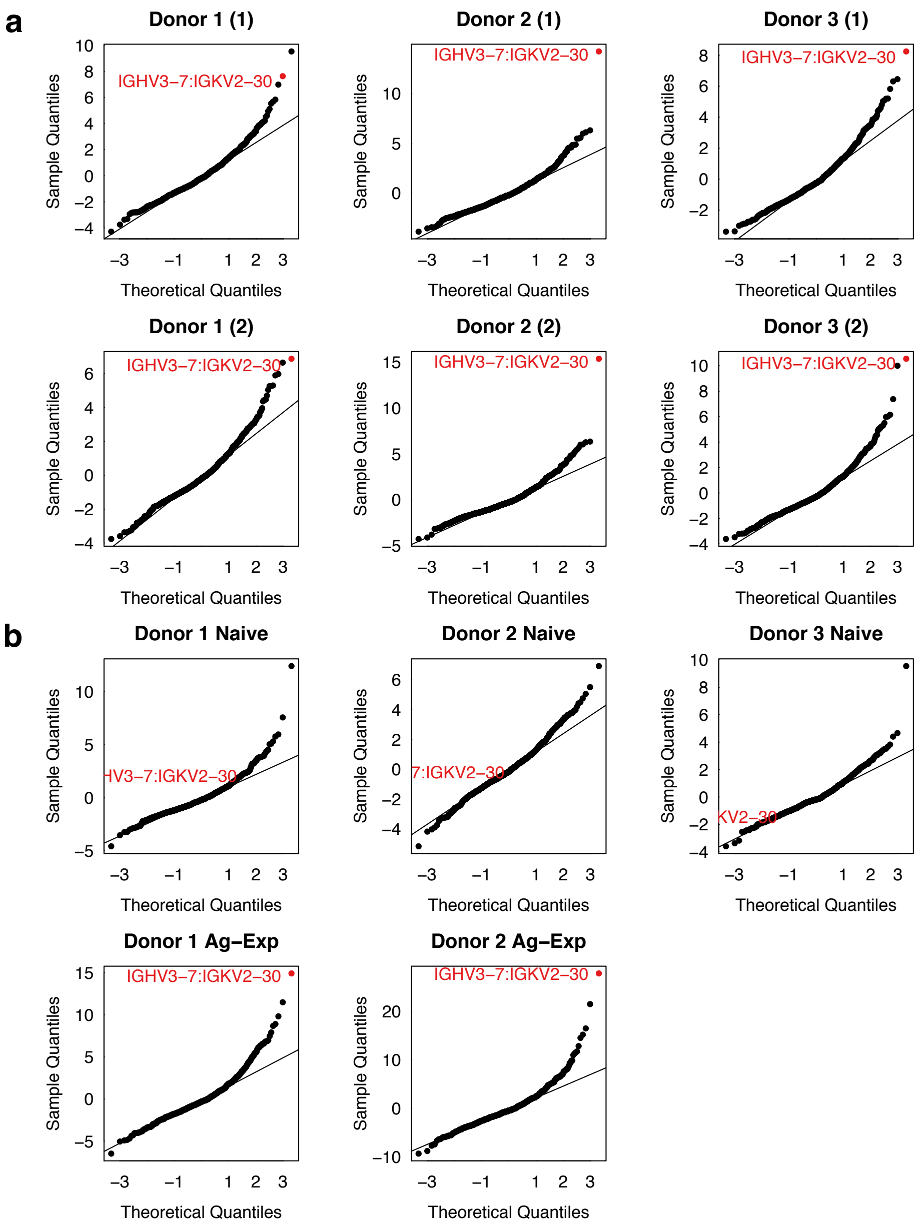

Supplementary Figure 10. QQ-plots of standardized residuals for V_H-V_L pairing frequencies. (a) Data from three human donors, each profiled at two different time points. (b) Data for naïve and antigen-experienced human B cells published in DeKosky, B.J. et al. Large-scale sequence and structural comparisons of human naive and antigen-experienced antibody repertoires. Proc Natl Acad Sci U S A 113, E2636-2645 (2016).

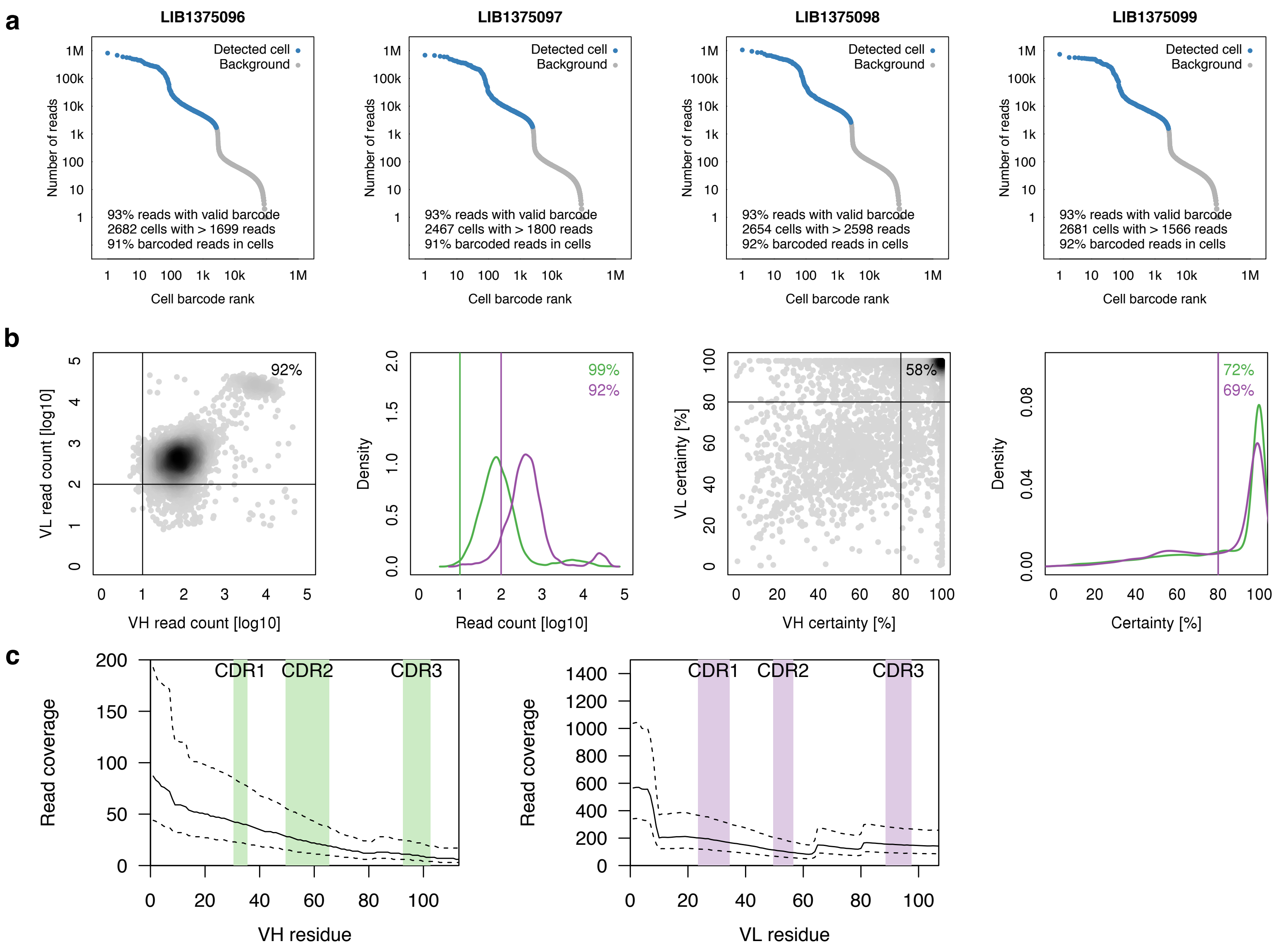

Supplementary Figure 11. Quality assessment of IgM^{neg}/OVA^{pos} lymph node B cell data. (a) Cell detection. (b) Cell quality filtering. (c) Read coverage for quality-filtered VH and VL assemblies.

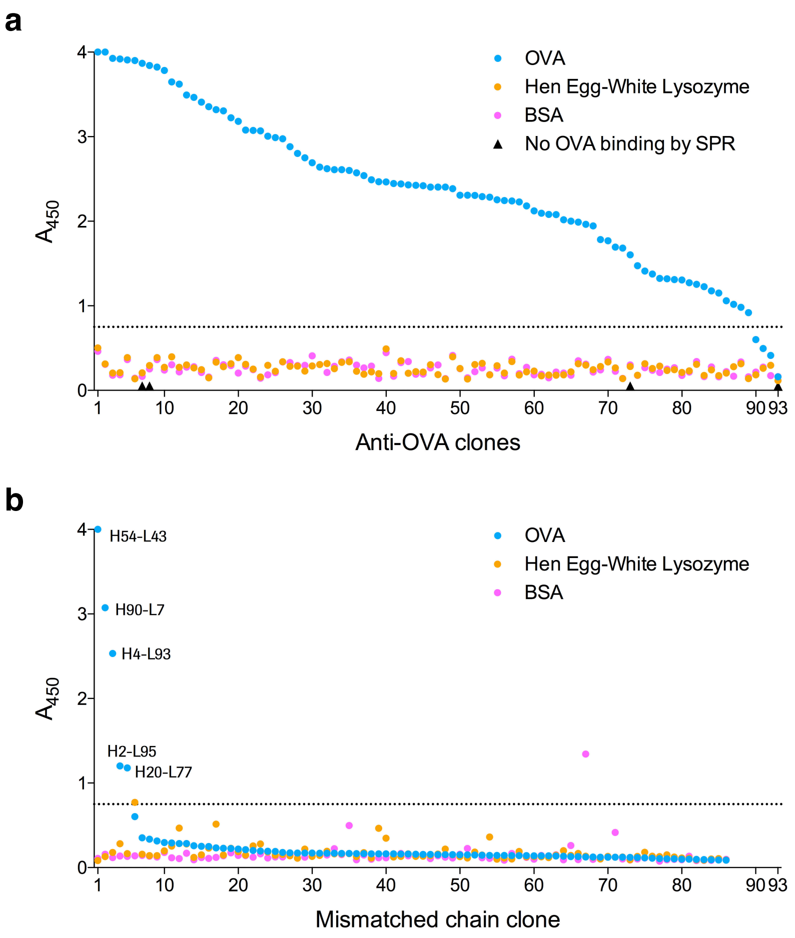

Supplementary Figure 12. ELISA results for predicted OVA antigen-reactive B cell clones (a) and negative controls obtained by mispairing VH and VL chains (b). Clones are ranked by OVA ELISA signal separately in (a) and (b).

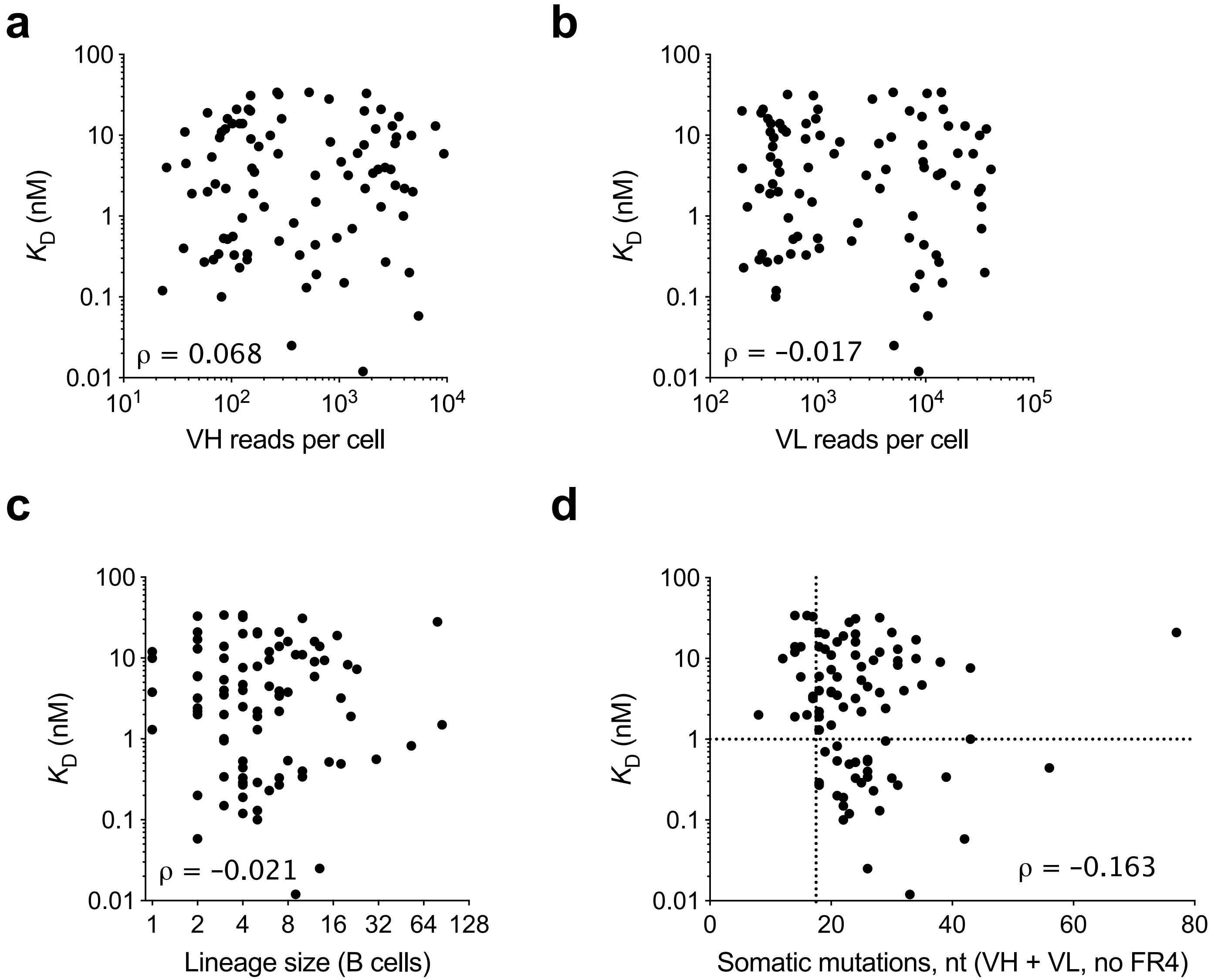

Supplementary Figure 13. Binding affinity (K_D) did not show obvious associations with VH read count (a), VL read count (b), lineage size (c), or SHM load (d).

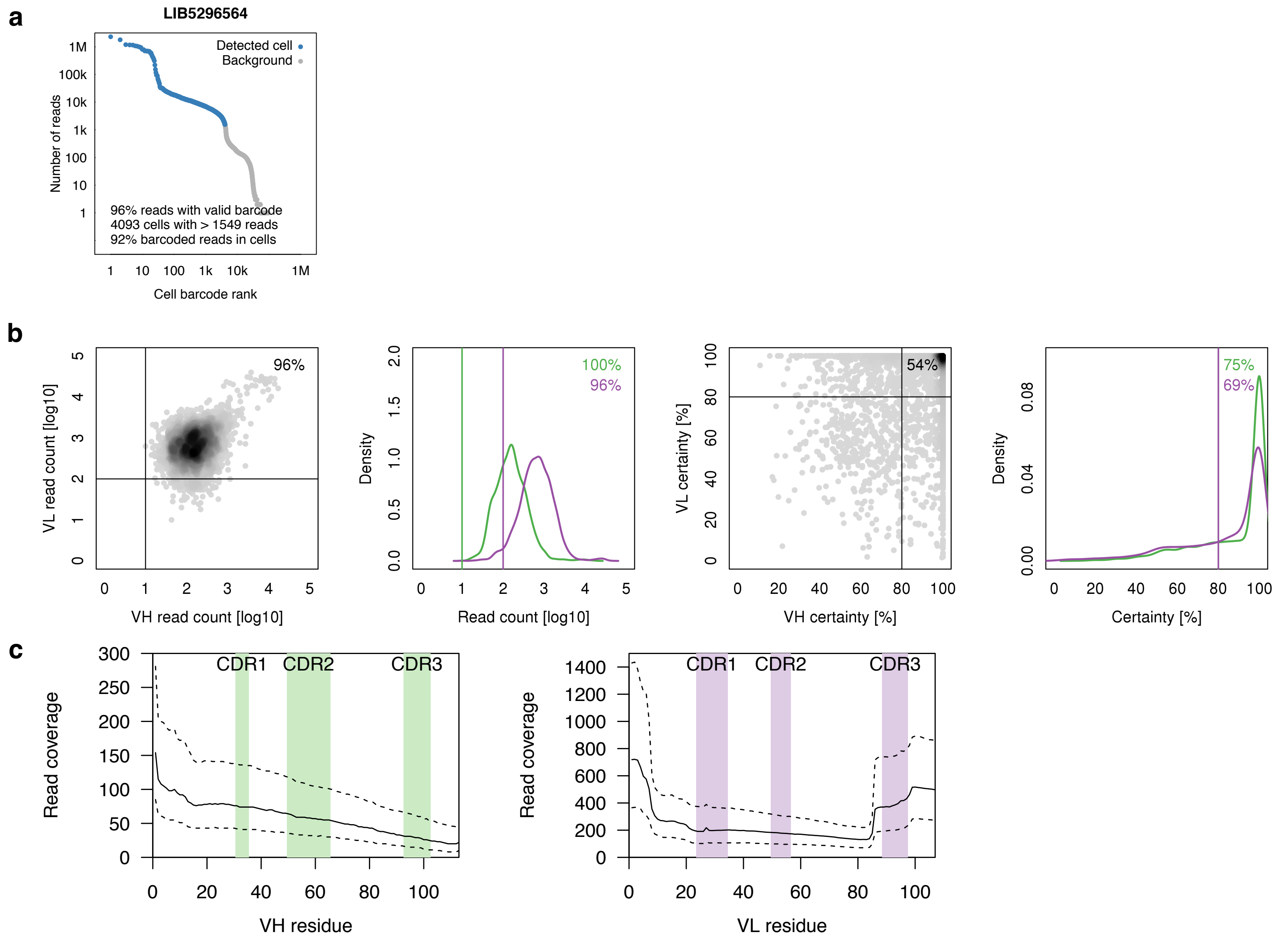

Supplementary Figure 14. Quality assessment of mouse data generated by 10x Genomics (B cells isolated from C57BL/6 mouse splenocytes - Direct Ig enrichment). (a) Cell detection. (b) Cell quality filtering. (c) Read coverage for quality-filtered VH and VL assemblies.

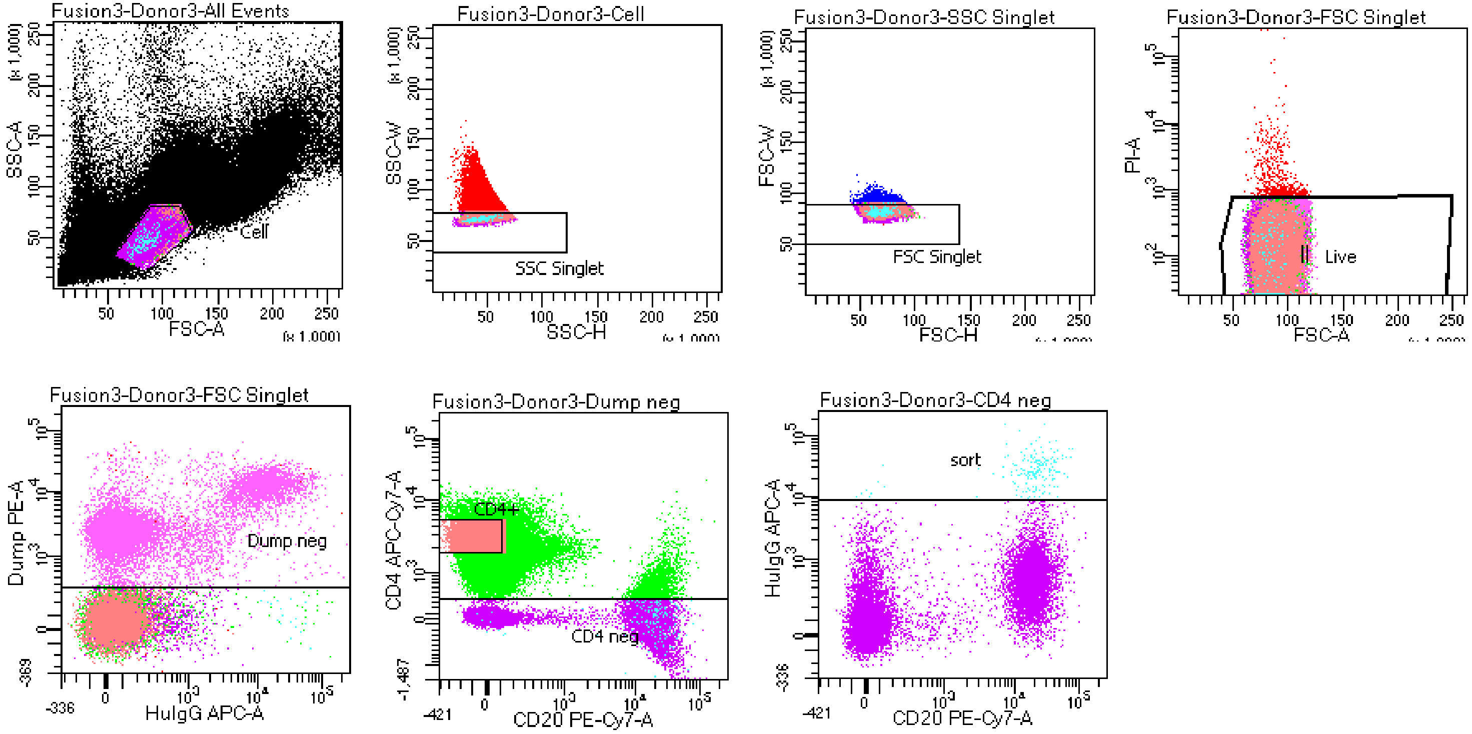

Tube: Donor3

Population	#Events	%Parent	%Total
All Events	510,250	####	100.0
Cell	250,147	49.0	49.0
SSC Singlet	233,951	93.5	45.9
FSC Singlet	231,564	99.0	45.4
Live	230,891	99.7	45.3
Dump neg	113,173	49.0	22.2
CD4 neg	11,722	10.4	2.3
sort	178	1.5	0.0
CD4+	49,323	43.6	9.7

Supplementary Figure 15. Human IgG^{pos} B cell sort gating strategy. 1) FSC vs SSC to gate lymphocytes, 2/3) SSC-H/SSC-W, FSC-H/FSC-W gates to exclude cell doublets, 4) FSC/PI for dead cell exclusion (PI-negative gate), 5) IgG-APC/Dump-PE (CD11b, CD11c, CD14, CD16, CD56, CD64, CD8) to exclude PE-positive non-B cells, 6) CD20-PE Cy7/CD4 APC-Cy7 to exclude CD4+ cells, 7) CD20-PECy7/IgG-APC to sort for IgG^{pos} B cells.

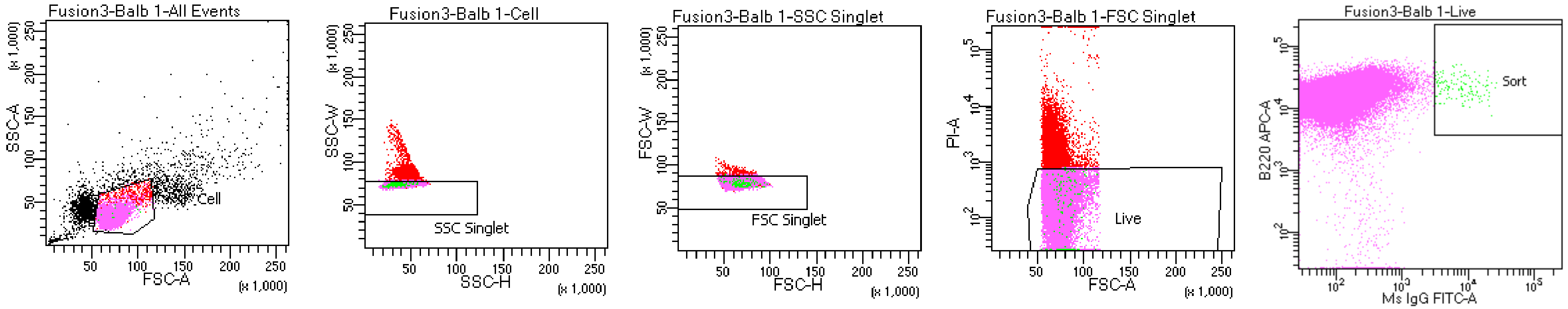

Tube: Balb 1

Population	#Events	%Parent	%Total
All Events	100,000	####	100.0
Cell	78,224	78.2	78.2
SSC Singlet	74,841	95.7	74.8
FSC Singlet	74,547	99.6	74.5
Live	69,670	93.5	69.7
Sort	141	0.2	0.1

Supplementary Figure 16. Naïve Balb/c mouse B cell sort gating strategy. 1) FSC vs SSC to gate lymphocytes, 2/3) SSC-H/SSC-W, FSC-H/FSC-W gates to exclude cell doublets, 4) FSC/PI for dead cell exclusion (PI-negative gate), 5) IgG FITC/B220-APC to sort for IgG^{pos} B cells.

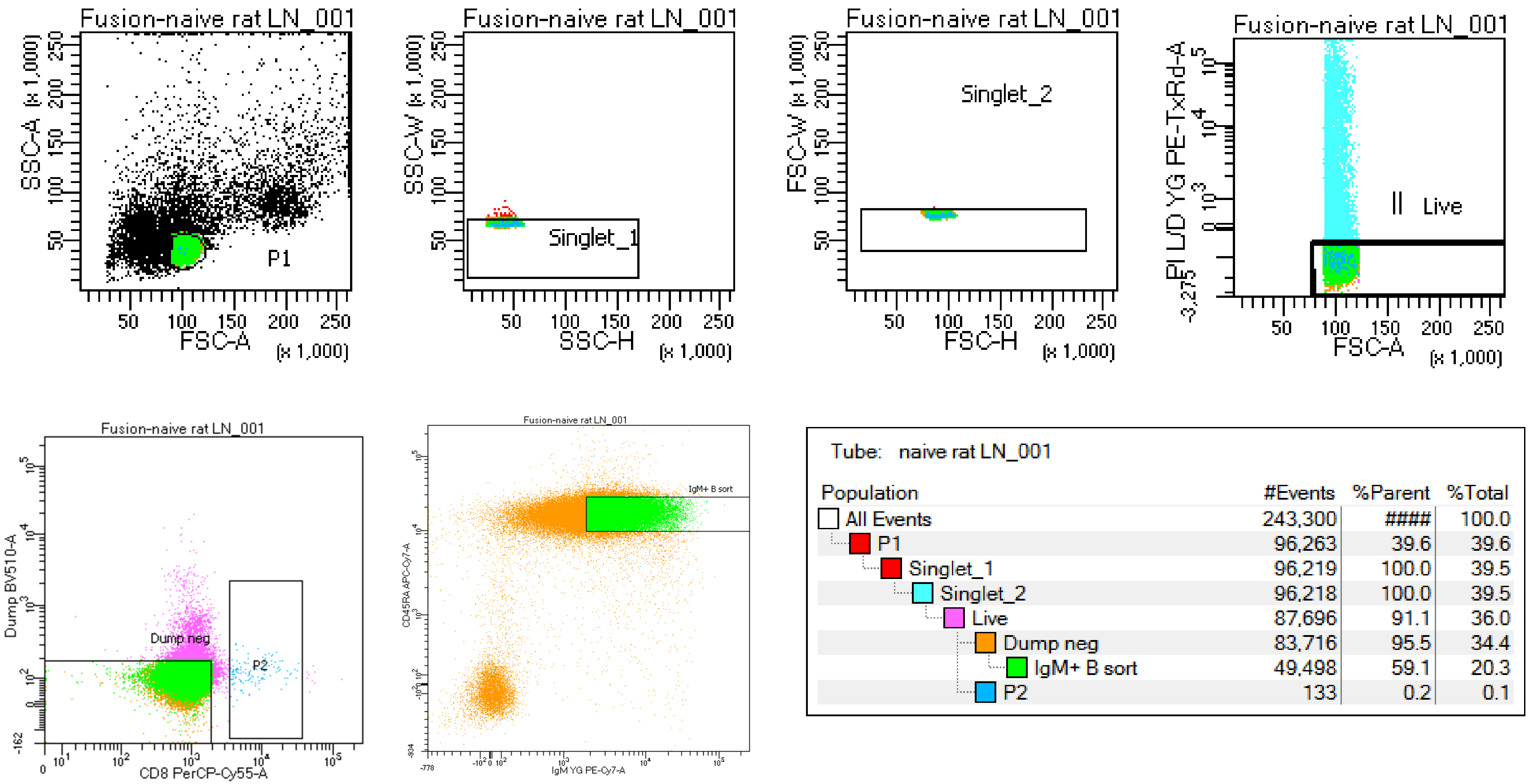

Supplementary Figure 17. Naïve SD rat B cell sort gating strategy. 1) FSC vs SSC to gate lymphocytes, 2/3) SSC-H/SSC-W, FSC-H/FSC-W gates to exclude cell doublets, 4) FSC/PI for dead cell exclusion (PI-negative gate), 5) CD8-PerCP Cy5.5/Dump-BV510 (CD4, CD11b, CD161a, granulocyte marker) to exclude CD8+BV510+ non-B cells, 6) IgM-PECy7/CD45RA-APC Cy7 to sort for B cells.

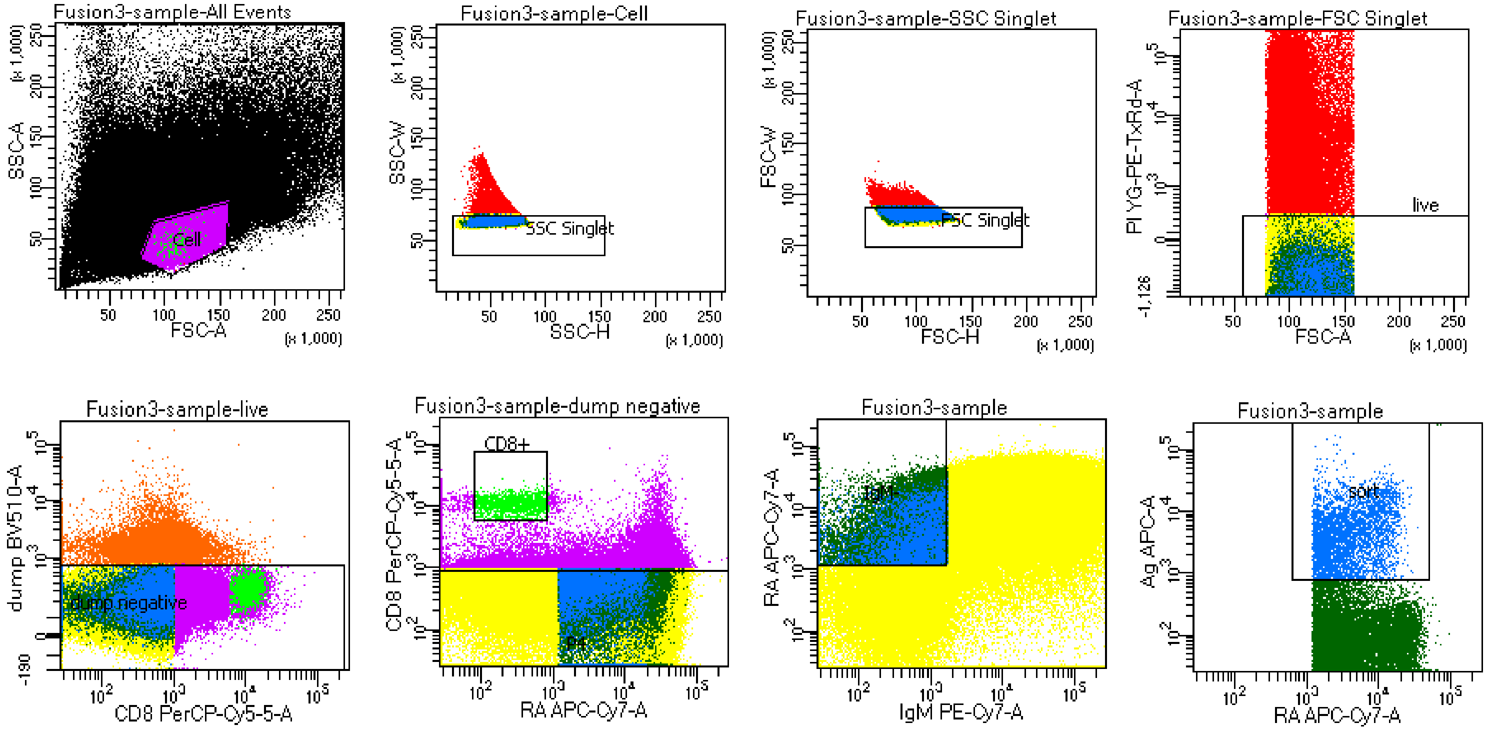

Tube: sample

Population	#Events	%Parent	%Total
All Events	4,225,000	####	100.0
Cell	1,328,095	31.4	31.4
SSC Singlet	1,304,252	98.2	30.9
FSC Singlet	1,287,995	98.8	30.5
live	1,194,887	92.8	28.3
dump negative	1,141,436	95.5	27.0
CD8+	1,391	0.1	0.0
P4	1,013,025	88.8	24.0
IgM-	39,402	3.9	0.9
sort	4,356	11.1	0.1

Supplementary Figure 18. Immunized SD rat, OVA^{pos}IgM^{neg} B cell sort gating strategy. 1) FSC vs SSC to gate lymphocytes, 2/3) SSC-H/SSC-W, FSC-H/FSC-W to exclude cell doublets, 4) FSC/PI for dead cell exclusion (PI-negative gate), 5) CD8-PerCP Cy5.5/Dump-BV510 (CD4, CD11b, CD161a, granulocyte marker) to exclude BV510+ non-B cells, 6) CD45R-APC Cy7/CD8-PerCP Cy5.5 to exclude CD8+ cells, 7) IgM-PE Cy7/CD45R-APC Cy7 to gate for IgM^{neg} B cells, 8) CD45R-APC Cy7/OVA-APC to sort for OVA^{pos}IgM^{neg} B cells.

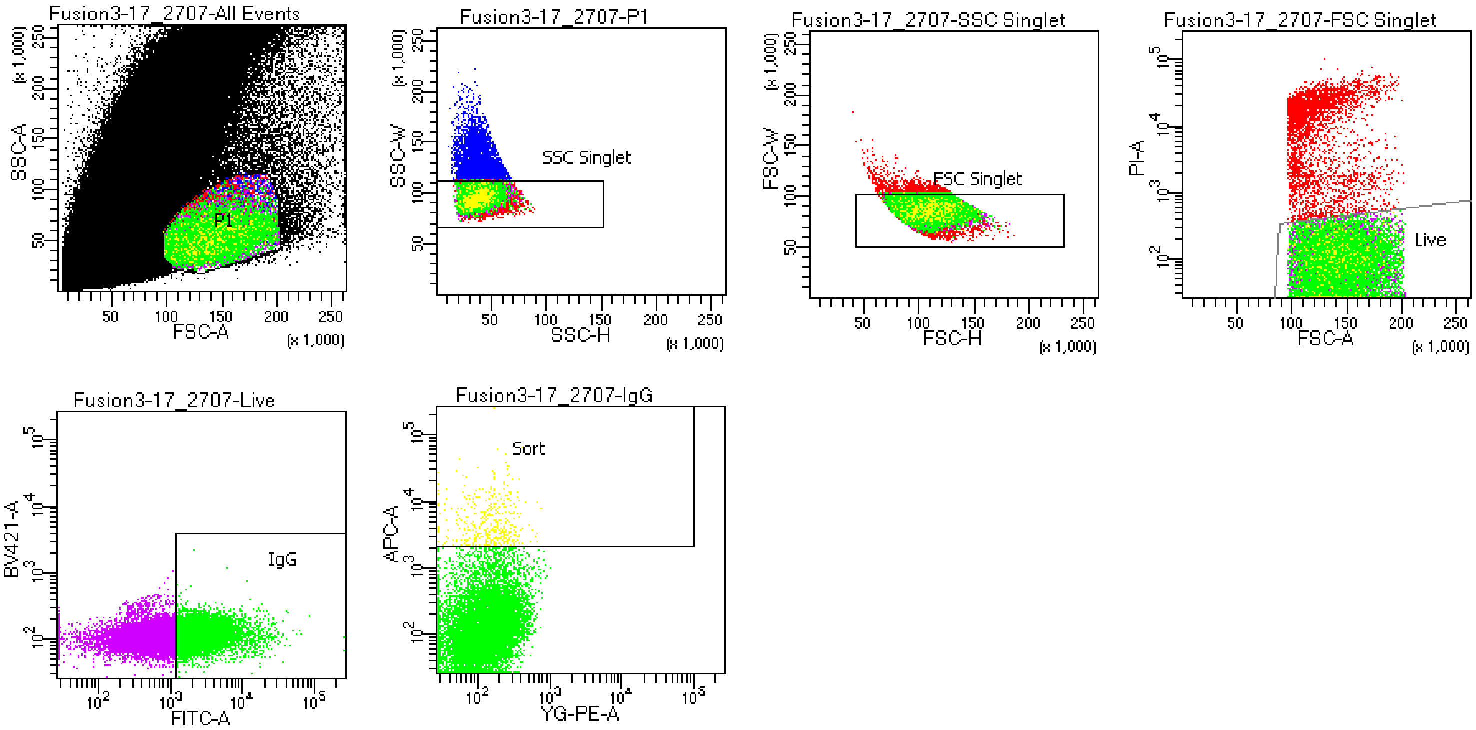

Tube: 17_2707

Population	#Events	%Parent	%Total
All Events	4,875,000	####	100.0
P1	36,297	0.7	0.7
SSC Singlet	32,718	90.1	0.7
FSC Singlet	31,846	97.3	0.7
Live	27,820	87.4	0.6
IgG	13,787	49.6	0.3
Sort	429	3.1	0.0
NOT(P1)	4,838,703	99.3	99.3

Supplementary Figure 19. Immunized SD rat, OVA^{pos}IgG^{pos} hybridoma sort gating strategy. 1) FSC vs SSC to gate hybridoma cells, 2/3) SSC-H/SSC-W, FSC-H/FSC-W gates to exclude cell doublets, 4) FSC/PI for dead cell exclusion (PI-negative gate), 5) IgG FITC/BV421 (empty channel) to gate for IgG^{pos} hybridomas, 6) PE (empty channel)/OVA-APC to sort for OVA^{pos}IgG^{pos} hybridomas.

PCR primers (reverse strand, constant region)

Rat CH1, outside PCR primer: YYTKGACCAGGCAKCCCAKDGTCAC
Rat CH1, inside PCR primer: CCAGGAGCCAGTGGATAGAC
Rat Cκ, outside PCR primer: AGGCACCTCCAGTTGCTAAC
Rat Cκ, inside PCR primer: CAGTTGGTGCAGCATCAGCCCG

Mouse CH1, outside PCR primer: GGGAARTAVYCCTTGACCAGGCABCC
Mouse CH1, inside PCR primer: GRCCARKGGATAGACHGATG
Mouse Cκ, outside PCR primer: GACTGAGGCACCTCCAGATG
Mouse Cκ, inside PCR primer: CTGCTCACTGGATGGTGGGAAG

List of additional Rat VH germlines not in IMGT database

>rIGHV2U2 ; wgs
CAGGTGCAGCTGAAGGAGTCAGGACCTGGTCTGGTGCAGCCCTCAGAGACCCTGTCCCTCACCTGCACTGTCTCTGGGTTCTCATT
AACCAGCAATAGTGTACACTGGGTTCGCCAGCCTCCAGGAAAGGGTCTGGAGTGGATGGGAATAATACGGAGTGGTGGAAGCA
CAGATTATAATTCAGCTCTCAAATCCCGACTGAGCATCAGCAGGGACACCTCCAAGAGCCAAGTTTTCTTAAAAATGAACAGTCTG
CAAACTGAAGACACAGCCATTTACTACTGTACCAGA

>rIGHV2U3 ; wgs
CAGGTGCAGCTGAAGGAGTCAGGACCTGGTCTGGTGCAGCCCTCACAGACCCTGTCCCTCACCTGCACTGTCTCTGGATTCTCATT
AACCAGCTATCATGTAAGCTGGGTTTGCCAGCCTCCAGGAAAGGGTCTGGAGTGGATGGGAAGAATACAGAGTGGTGGAAGCAC
AGATTATAATTCAGCTCTCAAATCCCGACTGAGCATCAGCAGGGACACCTCCAAGAGCCAAGTTTTCTTAAAAATGAACAGTCTGC
AAACTGAAGACACAGCCATTTACTACTGTACCAGAGA

>rIGHV2U4 ; wgs
CAGGTGCAGCTGAAGGAGTCAGGACCTGGTCTGGTGCAGCCCTCACAGACTTTGTCTCTCACCTGCACTGTCTCTGGGTTCTCACT
AACCAGCTATCATGTAAGCTGGGTTCGCCAGCCTCCAGGAAAAGGTCTGGAGTGGATGGGAGTAATATGGACTGGTGGAAGCAC
AGCATATAATTCACTTCTCAAATCCCGACTGAGCATCAGCAGGGACATCTCCAAGAGCCAAGTTTTCTTAAAAATGAACAGTCTGC
AAACTGAAGACACAGCCACTTACTACTGTGCCAGAGA

>rIGHV2U5 ; wgs
CAGGTGCAGCTGAAGGAGTCAGGACCTGGTCTGGTGCAGCCCTCACAGACCCTGTCTCTCACCTGCACTGTCTCTGGGTTCTCACT
AACCAGCTATCATGTAAGCTGGGTTCGCCAGCCTCCAGGAAAGGGTCTGGAGTGGATGGGAGGAATATGGGGTGATGGAAGCA
CAGCATATAATTCACTTCTCAAATCCCGACTGAGCATCAGCAGGGACACCTCCAAGAGCCAAGTTTTCTTAAAAATGAACAGTCAG
CAAACTGAAGACACAGCCATTTACTTCTGTACCAGA

>rIGHV2U6 ; wgs
CAGGTGCAGCTGAAGGAGTCAGGACCTGGTCTGGTGCAGCCCTCACAGACCCTGTCCCTCACCTGCACTGTCTCTGGGTTCTCACT
AAGCAGCTATGGTGTAATCTGGGTTCACCAGCCTCCAGGAAAGGGTCTGGAGTGGATGGGAGGAATATGGGGTGATGGAAGCA
CAGATTATAATTCAGCTCTCAAATCCCGACTGAGCATCAGCAGGGACACCTCCAAGAGTCAAGTTTTCTTAAAAATGAACAGTCTG
CAAACTGAAGACACAGCCATTTACTTCTGTACCAGA

>rIGHV2U8 ; wgs
CAGGTGCAGCTGAAGGAGTCAGGATCTGGTCTGGTGCAGCCCTCACAGACCCTGTCCCTCACCTGCACTGTCTCTGGGTTCTCACT
AACCAGCTATGGTGTAAGCTGGGTTCGCCAGCCTCCAGAAAAGGGTCGGGAGTGGATTGGAGCAATATGGACTGGTGGAAGCAC
AGATTATAATTCAGCTCTCAAATCCCGACTGAGCATCAGCAGGGACACCTCCAAGAGCCAAGTTCTCTTAAAAATGAACAGTCTGC
AAACTGAAGACACAGCCATGTACTTCTGTGCCAGAAA

>rIGHV2U9 ; wgs

CAGGTACAACTGAAGGAGACAGGACCTGACCTAGTGCAACTGACACAGACCCTGTCCATCACATGCACTGTCTCTGGGTTCTCATT
AACCACCTATAATGTTCACTGGGTCCGTCAGCCTCCAGGAAAGGGTCTGGAGTGGATGGGAGCAATGTGGAATGGTGGAGGCAC
AGATTATAATTCAGCATTTAAATCCCGACTGAGTATCAGCAGGGACACCTCCAAGAGCCAAGTTTTCTTAAAAATGAACAGTTTGC
AAACTGATGACACAGCCAAGTACTTCTGTGCCAGAA

>rIGHV2U12 ; wgs
CAGGTGCAGCTGAAGGAGTCAGGACCTGGTCTGGTGCAGCCCTCAGAGACCCTGTCCCTCACCTGCACTGTCTCTGGGTTCTCACT
AACCAGCTATAGTGTAAGTTGGGTTCGCCAGCCTTCAGGAAAAGGTCCTGAGTGGATGGGAAGAATGTGGTATGATGGAGACAC
AGCATATAATTCAGCTCTCAAATCCCGACTGAGCATCAGCAGGGACACCTCCAAGAACCAAGTTTTCTTAAAAATGAACAGTCTGC
AAACTGATGACACAGGCACTTACTACTGTACCAGAGA

>rIGHV2U14 ; wgs
CAGGTGCAGCTGAAGGAGTCAGGACCTGGCCTGGTGAAGCCCTCAGAGACCCTGTCTCTCACCTGCACTGTCTCTGGGTTCTCATT
AACCAGCTATCATGTAAGCTGGGTTCGACAGCCTCCAGGAAAGGGTCTGGAGTGGATGGGAGTAATATGGGGTGATGGAAGCAC
AGCATATAATTCAGCTCTCAAATCCCGACTGAGCATCAGCAGGGACACCTCCAAGAGCCAAGTTTTCTTAAAAATGAGCAGTCTGA
AAACTGAAGACACAGCCACTTACTACTGTGCCAGAGA

>rIGHV2U16 ; wgs
CAGGTGCAGCTGAAGGAGTCAGGACCTGGTCTGGTGCAGCCCTCACAGACCCTGTCCCTCACCTGCACTGTCTCTGGGTTCTCACT
AACCAGCTATGGTGTAAGCTGGGTTCGCCAGCCTCCAGGAAAGGGTCTGGAGTGGATGGGAGGAATATGGGGTGATGGAAGCA
CAAATTATAATTCAGCTCTCAAATCCCGACTGAGCATCAGCAGGGACACCTCCAAGAGCCAAGTTTTCTTAAAAATGAACAGTCTG
CAAACTGAAGACACAGCCATTTACTTCTGTACCAGAGA

>rIGHV5U21 ; wgs
GAGGTGCAGCTGGTGGAGTCTGGGGGAGGCTTAGTGCAGCCTGGAAGGTCCATGAAACTCTCCTGTGCAGCCTCAGGATTCACT
TTCAGTAACTATGACATGGCCTGGGTCCGCCAGGCTCCAAAGAAGGGTCTGGAGTGGGTCGCAACCATTAGTTATGATGGTAGTA

GCACTTACTATCGAGACTCCGTGAAGGGCCGATTCACTATCTCCAGAGATAATGCAAAAAGCACCCTATACCTGCAAATGGACAGT
CTGAGGTCTGAGGACACGGCCACTTATTACTGTACAACAGA

>rIGHV5U22 ; wgs
GAGGTGCAGCTGGTGGAGTCTGGGGGAGGCTTAGTGCAGCCTGGAAGGTCCATGAAACTCTCCTGTGCAGCCTCTGGATTCACTT
TCAGTAGCTTTGCAATGGCCTGGGTCCGCCAGGCTCCAACGAAGGGGCTGGAGTGGGTCGCATCCATTAGTTATGATGGTGGTAA
CACTTACTATCGAGACTCCGTGAAGGGCCGATTCACTATCTCCAGAGATAATGCAAAAAGCAGCCTATACCTGCAAATGGACAGTC
TGAGGTCTGAGGACACGTCCACTTATTACTGTGCAAAAGACA

>rIGHV5U23 ; wgs
GAGGTGCAGCTGGTGGAGTCTGGGGGCGGCTTAGTGCAGCCTGGAAGGTCCATGAAACTCTCCTGTGCAGCCTCAGGATTCACT
TTCAGTAACTATGGCATGGCCTGGGTCCGCCAGGCTCCAAAGAAGGGTCTGGAGTGGGTCGCATACATTAGTTATGATGGTGGTA
GCACTTACTATCGAGACCCCGTGAAGGGCCGATTCACTATCTCCAGAGATAATGCAAAAAGCACCCTATACCTGCAAATGGACAG
TCTGAGGTCTGAGGACACGGCCACTTATTACTGTACAACAGA

>rIGHV5U25 ; wgs
GAGGTGCAGCTGGTGGAGTCTGGGGGAGGATTAGTGCAGCCTGGAAGGTCCCTGAAACTCTCCTGCGCAGCCTCAGGATTCACT
TTCAGTAGCTTTCCAATGGCCTGGGTCCGCCAGGCTCCAAAGAAGGGTCTGGAGTGGGTCGCATCCATTAGTTCTGGTGGTGGTG
GCACTTACTATCCAGATTCCGTGAAGGGCAGATTTACTATCTCCAGAGATAATGCAAAAAGCACCCTATACCTGCAAATGGACAGT
CTGAGATCTGAGGACACGGCCAGTTACTATTGTGCAAGACA

>rIGHV2U17d ; repertoire NGS
CAGGTGCAGCTGAAGGAGTCAGGACCTGGTCTGGTGCAGCCCTCACAGACCCTGTCCCTCACCTGCACTGTCTCTGGGTTCTCATT
AACCAGCAATAGTGTACACTGGGTTCGCCAGCCTCCAGGAAAGGGTCTGGAGTGGGTTGGAGCAATATGGAGTGGTGGAAGCAC
AGATTATAATTCAGCTCTCAAATCCCGACTGAGCATCAGCAGGGACACCTCCAAGAGCCAAGTTTTCTTAAAAATGAACAGTCTGC
AAACTGAAGACACAGCCATTTACTTCTGTACCAGA

>rIGHV2U18d ; repertoire NGS
CAGGTGCAGCTGAAGGAGTCAGGACCTGGTCTGGTGCAGCCCTCACGGACCCTGTCCCTCACCTGCACTGTCTCTGGGTTCTCGCT
CACCAGCTATGGTGTAAGCTGGGTTCGCCAGCCTCCAGGAAAGGGTCTGGAGTGGATTGCAGCAATATGGAGTGGTGGAAGTAC
AGATTATAATTCAGCTCTCAAATCCCGACTAAGCATCAGCAGGGACACCTCCAAGAGCCAAGTTCTCTTAAAAATGAACAGTCTGC
AAACTGAAGACACAGCCATGTACTTCTGTGCCAGA

>rIGHV3U1d ; repertoire NGS
GAGGTGCAGCTTCAGGAGTCAGGACCTGGCCTTGTGAAACCCTCACAGTCACTCTCCCTCACCTGTTCTGTCACTGGTTACTCCATC
ACTAGTAATTACTGGGGCTGGATCCGGAAGTTCCCAGGAAATAAAATGGAGTGGATGGGATACATAAGCTACAGTGGTAGCACT
AGCTACAACCCATCTCTCAAAAGTCGAATCTCCATTACTAGAGACACATCGAAGAATCAGTTCTTCCTGCAGTTGAACTCTGTAACT
ACTGAGGACACAGCCACATATTACTGTGCAAGA

>rIGHV3U2d ; repertoire NGS
GAGATACAGCTGCAGGAGTCAGGACCTGGCCTTGTGAAACCTTCACAGTCACTCTCCCTCACCTGTTCTGTCACTGGTTACACCATT
ACCAGTGGTTATGATTGGAGCTGGATCCGGAAGTTCCCAGGAAATAAAATGGAGTGGATGGGATACATAAGCTACAGTGGTAGC
ACTAACTACAACCCATCGCTCAAAAGTCGAATCTCCATTACTAGAGACACATCCAAGAATCAGTTCTTCCTGCAGTTGAACTCTGTA
ACTACTGAGGATACAGCCACATATTACTGTGCAAGA

>rIGHV5U27d ; repertoire NGS
GAGGTGCAGCTGGTGGAGTCTGGCGGAGGCTTAGTACAGCCTGGAAGGTCCCTGAAACTCTCCTGTGCAGCCTCAGGATTCACTT
TCAGTAACTATTACATGGCCTGGGTCCGCCAGGCTCCAACGAAGGGTCTGGAGTGGGTCGCATCCATTACTAATAGTGGTGGTAG
TACTTACTATCGAGACTCCGTGAAGGGCCGATTCACTATCTCCAGAGATAATGCAAAAAGCACCCTATACCTGCAAATGGACAGTC
TGAGGTCTGAGGACACGGCCACTTATTACTGTACAAGA

>rIGHV5U28d ; repertoire NGS
GAGGTGCAGCTGGTGGAGTCTGGGGGCGGCTTGGTGCAGCCTGGAAGGTCCCTGAAACTCTCCTGTGCAGCCTCAGGATTCACT
TTCAGTAACTATGACATGGCCTGGGTCCGCCAGGCTCCAACGAAGGGTCTGGAGTGGGTCGCATCCATTAGTCCTAGTGGTGGTA

GCACTTACTATCGAGACTCCGTGAAGGGCCGATTCACTATCTCCAGAGATAATGCAAAAAGCACCCTATACCTGCAAATGGACAGT
CTGAGATCTGAGGACACCGCCACTTATTACTGTGCAACG

>rIGHV5U29d ; repertoire NGS
GAGGTGCAGCTGGTGGAGTCTGGGGGAGGCTTAGTGCAGCCTGGAAGGTCCCTGAAACTCTCCTGTGCAGCCTCAGGATTCACT
TTCAGTGACTATAACATGGCCTGGGTCCGCCAGGCTCCAACGAAGGGTCTGGAGTGGGTTGCATCCATTAGTTATGGTGGTAGTA
ACACCCACTATGGAGACTCCGTGAAGGGCCGATTCACTATCTCCAGAGATAATGCAAAAAGCACCCTATACCTGCAAATGGACAG
TCTGAGGTCTGAGGACACGGCCACTTATTACTGTGCAAGA

>rIGHV5U30d ; repertoire NGS
GAGGTGCAGCTGGTGGAGTCTGGGGGAGGCCTAGTGCAGCCTGGAAGGTCCCTGAAACTCTCCTGTGCAGCCTCAGGATTCACT
TTCAGTAACTATTACATGGCCTGGGTCCGCCAGGCTCCAAAGAAGGGTCTGGAGTGGGTCGCAACCATTAGTACCAGTGGTAGCA
GAACTTACTATCCAGACTCCGTGAAAGGCCGATTCACTATCTCCAGAGATAATGCAAAAAGCAGCCTATACCTGCAAATGAACAGT
CTGAAGTCTGAGGACACGGCCACTTATTACTGTGCAAGA

>rIGHV5U31d ; repertoire NGS
GAGGTGCAGCTGGTGGAGTCTGGGGGAGGCTTAGTGCAGCCTGGAAGGTCCATGAAACTCTCCTGTGCAGCCTCAGGATTCACT
TTCAATAACTATGACATGGCCTGGGTCTGCCAGGCTCCAAAGAAGGGTCTGGAGTGGGTCGCAACCATTAGTTATGATGGTAGTA
GCACTTACTATCGAGACTCCGTGAAGGGCCGATTCACTATCTCCAGAGATAATGCAAAAAGCACCCTATACCTGCAAATGGACAGT
CTGAGGTCTGAGGACACGGCCACTTATTACTGTGCAAGA

List of additional Rat Vκ germlines not in IMGT database

>rIGKV1U5 ; wgs
GATGTTGTGATGACCCAGACACCACCATCTTTGTCGGTTGCCATTGGACAGTCAGTCTCCATCTCTTGCAAGTCAAGTCAAAGCCTC
GTAGCTAGTGATAAAAATACATATTTGAATTGGTTATTACAGAGTCCTGGCCGGTCTCCGAAGCGCCTAATCTATCAGGTGTCTAA

GCTGGACTCTGGAGTCCCTGACAGGTTCAGTGGCAGTGGATCAGAGAAAGATTTCACACTTAAAATCAGCAGAGTGGAGGCTGA
GGATTTGGGAGTTTATTACTGCCTGCAAGGTACACATCTTCCTCA

>rIGKV1U10 ; wgs
GATGTTGTGTTGACACAAACTCCAGTTGCCCAGCCTGTCACACTTGGAGATCAAGCTTCTATATCTTGCAGGTCTAGTCAGAGCCT
GGTACATAGTAATGGAAACACTTATTTGGAATGGTACCTACAGAAGCCAGGCCAGTCTCCACAGCTCCTCATCTATAAGGTTTCCA
ACCGATTTTCTGGGGTACCAGACAGGTTCATTGGCAGTGGGTCAGGGTCAGATTTCACCCTCAAGATCAGCAGAGTAGAGCCTGA
GGACTTGGGAGTTTATTACTGCTTCCAAGCTACACATGATCCTCC

>rIGKV1U75 ; wgs
GATGTTGTGTTGACACAAACTCCAGGTTCCCTGTCTGTCACACTTGGAGATCAAGCTTCTATATCTTGCAGGTCTAGTCAGAGCCTG
GAATATAGTGATGGATACACTTATTTGGAATGGTACCTACAGAAGCCAGGCCAGTCTCCACAGCTCCTCATCTATGAAGTTTCCAA
CCGATTTTCTGGGGTCCCAGACAGGTTCATTGGCAGTGGGTCAGGGACAGATTTCACCCTCAAGATCAGCAGAGTAGAGCCTGAG
GACTTGGGAGTTTATTACTGCTTCCAAGCTACACATGATCCTCC

>rIGKV2U3 ; wgs
GATGTTGTGCTGACCCAGACTCCACCCACTTTATCGGCTACCATTGGACAATCGGTCTCCATCTCTTGCAGGTCAAGTCAGAGTCTC
TTAGATAGTGATGGAGATACCTATTTAAATTGGTTGCTACAGAGGCCAGGCCAATCTCCACAGCTTCTAATTTATTCGGTATCCAAC
CTGGAATCTGGGGTCCCCAACAGGTTCAGTGGCAGTGGGTCAGAAACAGATTTCACACTCAAAATCAGTGGAGTGGAGGCTGAA
GATTTGGGAGTTTATTACTGCATGCAAGCTACCCATGCTCCTCT

>rIGKV2U13 ; wgs
GATATTGTGATGACTCAAGCTCCACTCTCTGTATCTGTCACTCCTGGAGAGTCAGCTTCCATCTCCTGCAGATCTAGTAAGAGTCTG
CTAAGTAGTAAGGGCATCACTTCCTTGTATTGGTACCTTCAGAGGCCAGGAAAGTCTCCTCAGCTCCTGATATATCGGATGTCCAA
CCTTGCCTCAGGAGTTCCAGACAGGTTTAGTGGCAGTGGGTCAGAAACCGATTTTACACTGAAAATCAGTAAGGTGGAGACTGAG
GATGTTGGTGTTTATTACTGTGGACATCGGTCTAGAATATCCTCC

>rIGKV2U17 ; wgs
GATATTGTGATGACCCAGGGTGCACTCCCCAATCCTGTCCCTTCTGGAGAGTCAGCTTCCATCACCTGCCAGTCTAGTAAGAGTCT
GCTGCACAGCAATGGCAAGACATACTTGAATTGGTATCTGCAGAGGCCAGGACAGTCTCCTCAGCTCCTGATCTATTGGATGTCTA
CCCGTGCATCAGGAGTCTCAGACAGGTTCAGTGGCAGTGGGTCAGGAACAGATTTCACACTGAAAATCAGTAGCGTGGAGGCTG
AGGATGTGGGTGTGTATTACTGTCAGCAATTTCTAGAGTATCCTC

>rIGKV2U18 ; wgs
GATATTGTGTTGACTCAAGCTCCACTCTCTGTATCTGTCACTCCTGGAGAGTCAGCTTCCATCTCCTGCAGGTCTAGTAAGAGTCTG
CTAAGTAGTAAGGGCATCACTTCCTTGTATTGGTACCTTCAGAGGCCAGGAAAGTCTCCTCAGCTCCTGATATATCAGATGTCCAA
CCTTGCCTCAGGAGTTCCAGACAGGTTTAGTAGCAGTGGGTCAGAAACAGATTTTACACTGAAAATCAGTAAGGTGGAGACTGAG
GATGTTGGTGTTTATTACTGTGGACATCGTCTAGAATATCCTCC

>rIGKV3U19 ; wgs
GACATTGTGCTGACCCAGTCTCCTGCTTTGGCTGTGTCTCTAGAGCAGAGAGCCACCATCTCTTGCAAAACCAGCCAGAATGTCGA
TAATTATGGCATTAGTTATATGCACTGGTACCAACAGAAACCAGGACAGCAACCCAAACTCCTCATCTATGGTGCATCCAACCTAG
AGTCTGGAGTCCCTGCCAGGTTCAGTGGCAGTGGGTCTGGGACAGACTTCACCCTCACCATCGATCCTGTGGAGGCTGATGATAT
TGCAACCTATTACTGTCAGCAGAGTAAGGATTATCCTCC

>rIGKV4U34 ; wgs
GAAATTGTGCTAACCCAGTCTCCAACAACCATGGCTGCATCTCCGGGGGAGAAGGTCACCATCACCTGCCGTGCCAGCTCCAGTG
TAAGCTACATGTACTGGTACCAGCAGAAGTCAGGCGCCTCCCCTAAACCCTGGATTTATGAAACATCCAAACTGGCTTCTGGAGTC
CCAGATCGCTTCAGTGGCAGTGGGTCTGGGACCTCTTATTCGTTCACAATCAGCTCCATGGAGACTGAAGATGCTGCCACTTATTA
CTGTCACCAGTGGAGTAGTACCCCACCCA

>rIGKV8U28 ; wgs
GACATTGTGATGACCCAGACTCCATCCTCCCAGGCTGTGTCAGCAGGGGAGAAGGTCACTATGAGCTGCAAGTCCAGTCAGAGTC
TTTTATACAGTGGAGACCAAAAGAACTACTTGGCCTGGTACCAGCAGAAACCTGGGCAGTCTCCTAAACTGCTGATCTACTTGGCA

TCCACTAGGGAATCAGGGGTCCCTGATCGCTTCATAGGCAGTGGATCTGGGACAGACTTCACTCTGACCATCAGCAGTGTGCAGG
CTGAAGATCTGGCAGATTATTACTGTCAGCAGCATTACAGCTATCCTCC

>rIGKV8U29 ; wgs
GACATTGTGATGACCCAGTCTCCCTCCTCCCTGGCTGTGTCAGCAGGAGACAAGGTCACTATGAGCTGCAAGTCCAGTCAGAGTCT
TTTATCCAGTGAATACCAAGGGAACTACTTGTCCTGGTTCCAGCAGAAACCAGGGCAGTCTCCTAAACTGCTGATCTCCTTGGCAT
CCACTAGGGAAACTGGTGTCCCTGATCGCTTCATAGGCAGTGGATCTGGGACAGACTTCACTCTGACCATCAGCAGTGTGCAGGC
TGAAGACCTGGCTGTTTATTACTGTGAGCAGTACTACAGCTATCCTCC

>rIGKV8U40 ; wgs
ACATTGTGATGACCCAGACTCCATCCTCCCAGGCTGTGTCAGCAGGGGAGAAGGTCACTATGAGCTGCAAGTCCAGTCAGAGTCT
TTTATACAGTGAAAACAAAAAGAACTACTTGGCCTGGTACCAGCAGAAACCAGGGCAGTCTCCTAAACTGCTGATCTACTGGGCA
TCCACTAGGGAATCTGGGGTCCCTGATCGCTTCATAGGCAGTGGATCTGGGACAGATTTCACTCTGACCATCAGCAGTGTGCAGG
CAGAAGACCTGGCTGTTTATTACTGCCAGCAGTACTATAACTTTCCTCC

>rIGKV10U55 ; wgs
GACATCAAGATGACCCAGTCTCCCTCCTCCCTGTCTGCATCTCTGGGAGAAAGAGTCACCATCAGTTGCAGGGCAAGTGAGAATAT
TAACAATATTTTGGCCTGGTATCAGAAGAAAGAAGATGGAAGTGTTAAACTCCTGATTTACTACACATCAAATCTACAATCTGGGG
TCCCATCAAGGTTCAGTGGCAGTGGGTCTGGGAAAGATTACTCTCTTACCATTAGTGGCCTAGAATCTGAAGATATTGCGACTTAC
TATTGTCAGCAAGGTTATACCCCACC

>rIGKV12U30 ; wgs
GACATCCAGATGACACAGTCTCCAGCTTCCCTGTCTGCATCTCTGGGAGAAACTGTCACCATCGAATGTCGAGCAAGTGAGGACAT
TTACAGTAATTTAGCGTGGTATCAGCAGAAACCAGGGAACTCTCCTCAGCTCCTGATCTATGATGCAAATAGCTTGGCAGATGGG
GTCCCATCACGGTTCAGTGGCAGTGGATCTGGCACACAGTATTCTCTAAAGATAAACAGCCTGCAATCTGAAGATGTCGCAAGTTA
TTTCTGTCAACAGTATAACAATTATCCTCC

>rIGKV12U74 ; wgs
GACATCCAGATGACACAGTCTCCAGCTTCCCTGTCTGCATCTCTGGGAGAAACTATCTCCATCGAATGTCTAGCAAGTGAGGGCAT
TTCCAGTTATTTAGCGTGGTATCAGCAGAAGCCAGGGAAATCTCCTCAGCTCCTGATCTATGGTGCAAATAGCTTGCAAGCTGGGG
TCCCATCACGGTTCAGTGGCAGTGGATCTGGCACACAGTATTCTCTCAAGATCAGCAGCATGCAACCTGAAGATGAAGGGGATTA
TTTCTGTCAACAGAGTTACAAGTTTCCTCC

>rIGKV12U76 ; wgs
GACATCCAGATGACACAGTCTCCTGCCTCCCTGTCTGCATCTCTGGAAGAAATTGTCACCATCACATGTCAGGCAAGCCAGGACAT
TGGTAATTGGTTGGCATGGTATAAGCAGAAACCAGGGAAATCTCTTCAGCTCCTGATCAATGATGCAACCAGCTTGGCAGACGGG
GTCCCATCAAAGTTCAGAGGCAGTAGATCTGGTACACAGTATTCTCTTAAGATCAGCAGACTACAGGATGAAGATTTTGGAAGCT
ATTACTGTCAACAGGCTCATAGTAATCCTCC

>rIGKV12U78 ; wgs
GACATCCAGATGACACAGTCTCCTGCCTCCCTGTCTGCTTCTCTGGAAGAAATTGTCACCATCACCTGCAAGGCAAGCCAGGGCAT
TGATGATTACTTATCATGGTATCAGCAGAAACCAGGGAAATCTCCTCAGCTCCTGATCTATGATGCAACCAGCTTGGCAGATGGGG
TCCCATCACGGTTCAGCGGCAGTAGATCTGGCACACAGTATTCTCTTAAGATCAGCAGACCACAGGTTGATGATTCTGGAATCTAT
TACTGTCTACAGAGTTACAGTACTCCTCC

>rIGKV12U81 ; wgs
GACATCCAGATGACACAGTCTCCTGCCTCCCTGTCTGCATCTCTGGAAGAAATTGTCACCATCACATTCCAGGCAAGCCAAGACAT
TGGTAATTGGTTGGCATGGTATCAGCAGAAACCGGGGAAATCTCCTCAGCTCCTGATTTATGATGCAACCAGCTTGGCAGATGGG
GTCCCATCACGGTTCAGCGGCAGTAGATCTGGCACACAGTATTCTCTTAAGATCAGCAGACTACAGGTTGAAGATATTGGAAGCT
ATTACTGTCAACAGGCTCATAGTAATCCTCC

>rIGKV15U51 ; wgs
GACATCCAGATGACCCAGTCTCCTTCACTCCTGTCTGCATCTGTGGGAGACAGAGTCACTCTCAACTGCAAAGCAAGTCAGAATAT
TTATAAGAACTTAGAGTGGTATCAGCAAAAGCATGGAGAAGCTCCAAAACTCCTGATATATTATACAAACAATTTGCAAACGGGC

ATCTCATCAAGGTTCAGTGGCAGTGGATCTGGTACAGATTACACACTCACCATCAGCAGCCTGCAGCCTGAAGATGTTGCCACATA
TTACTGCTATCAGTATAACAGCGGGCCC

>rIGKV16U47 ; wgs
GATGTCCAGATGACCCAGTCTCCGTCTTATCTTGCTGCGTCTCCTGGAGAAAGTGTTTCCATCAGTTGCAAGGCAAGTAAGAGCAT
TAGCAATTATTTAGCCTGGTATCAACAGAAACCTGGGGAAGCAAATAAGCTTCTTATCTACTCTGGGTCAACTTTGCAATCTGGAA
CTCCATCGAGGTTCAGTGGCAGTGGATCTGGTACAGATTTCACTCTCACCATCAGAAACCTGGAGCCTGAAGATTTTGCAGTCTAC
TACTGTCAACAGTATTATGAAAAACCACATC

>rIGKV22U24 ; wgs
GACATCAAGATGACCCAGTCTCCTTCATTCCTGTCTGCATCTGTGGGAGACAGAGTCACTATCAACTGCAAAGCAAGTCAGAATAT
TAACAAGTACTTAAACTGGTATCAGCAAAAGCTTGGAGAAGCTCCCAAACTCCTGATATATAATACAAACAATTTGCAAACGGGCA
TCCCATCAAGGTTCAGTGGCAGTGGATCTGGTACTGATTTCACACTCACCATCAGCAGCCTGCAGCCTGAAGATGTTGCCACATAT
TTCTGCTTGCAGCATAATAGTAGGCCG

>rIGKV22U25 ; wgs
GACATCCAGATGACCCAGTCTCCTTCATTCCTGTCTGCATCTGTGGGAGAAAGAGTCACTCTCAGCTGCAGAGCAAGTCAGAATAT
TAACAAGTACTTAGACTGGTATCAGCAAAAGCTTGGAGAAGCTCCCAAACTCCTGATATATAATACAAACAATTTGCATACGGGCA
TCCCATCAAGGTTCAGTGGCAGTGGATCTGGTACAGATTACACACTCACCATCAGCAGCCTGCAGCCTGAAGATGTTGCCACATAT
TTCTGCTTGCAGCGTAATAGTTGGCCG

>rIGKV22U26 ; wgs
GACATCCAGATGACCCAGTCTCCTTCAGTCCTGTCTGCATCTGTGGGAGACAGAGTCACTCTCAACTGCAAAGCAAGTCAGAATAT
TAACAAGTACTTAAACTGGTATCAGCAAAAGCTTGGAGAAGCTCCCAAACTCCTGATATATAATACAAACAATTTGCAAACGGGCA
TCCCATCAAGGTTCAGTGGCAGTGGATCTGGTACAGATTACACACTCACCATCAGCAGCCTGCAGCCTGAAGATGTTGCCACATAT
TTCTGCTTTCAGCATAATAGTTGGCCC

>rIGKV22U27 ; wgs
GACATCCAGATGACCCAGTCTCCTTCACTCCTGTCTGCATCTGTGGGAGACAGAGTCACTATCAACTGCAAAGCAAGTCAGAATAT
TAACAAGTACTTAAACTGGTATCAGCAAAAGCTTGGAGAAGCTCCCAAACTCCTGATATATAATACAAACAATTTGCAAACGGGCA
TCCCATCAAGGTTCAGTGGCAGTGGATCTGGTACAGATTACACACTCACCATCAGCAGCCTGCAGCCTGAAGATTTTGGCACATAT
TTCTGCTTTCAGCATAATAGTTGGCCC

>rIGKV22U33 ; wgs
GACATCCAGATGACCCAGTCTCCTTCATTCCTGTCTGCAACTGTGGGAGACAGAGTCACTATCAACTGCAAAGCAAGTCAGAATAT
TAACAAGTACTTAAACTGGTATCAGCAAAAGCTTGGAGAAGCTCCCAAACGCCTGATATATAATACAAACAGTTTGCAAACTGGCA
TTCCATCAAGGTTCAGTGGCAGTGGATCTGGTACAGATTACACACTCACCATCAGCAGCCTGCAGCCTGAAGATGTTGCTACATAT
TTCTGCTTGCAGCATAATAGTGGGCCC

>rIGKV22U53 ; wgs
AACATCCAGCTGACCCAGTCTCCTTCACTCCTGTCTGCATCTGTGGGAGACAGAGTCACTCTTAGCTGCAAAGGAAGTCAGAATAT
TAACAATTACTTAGCCTGGTACCAACAAAAGCTTGGAGAAGCTCCCAAACTCCTGATATATAATACAAACAGTTTGCAAACGGGCA
TCCCATCAAGGTTCAGTGGCAGTGGATCTGGTACAGATTACACACTCACCATCAGCAGCCTGCAGCCTGAAGATGTTGCCACATAT
TTCTGCTATCAGTATAACAACGGGTAC

>rIGKV22U72 ; wgs
GACATCCAGATGACCCAGTCTCCTTCACTCCTGTCAGCATCTGTGGGAGACAGAGTCACTCTCAGCTGCAAAGCAAGTCAGAGTAT
TTACAGCAGCTTAGCCTGGTATCAGCAAAAGCTTGGAGAAGCTCCCAAACTCCTGATATATAGTGCAAACAGTTTGCAAACGGGC
ATCCCGTCAAGGTTCAGTGGCAGTGGATATGGTACAGATTTCACACTCACCATCAGCAGCCTGCAGCCTGAAGATGTTGCCACATA
TTTCTGCCATCAGTATTACAGTTGGCCC

>rIGKVxU54 ; wgs
GATATTCAAGTGACTCAATCTCCATCCTCCCTCTTGGCATCTCTAGGAGAGAGAGTCACTATCACATGCCAGACAAGTCAGAGCAT
TAGCAATAACCTAAACTGGTATCAGCAGAAACCAGGACAAGCTCCTATGCTCTTGATCTATTATGCAACCAGTTTGCAGACTGGCA

TGCCATCAAGGTTCAGTGGCCAATATTCTGGGAGAAGTTTCACTCTAACCATCACTAGCCTGGAGCCAGAAGATATTGCAAATTAT
TTTTGTCTGCAGCATTACAGTGCTCCTCA

>rIGKVxU84 ; wgs
GATATCCAGATGAACCAGACTCCATCTACTCTATCTATGTCTATTGGAGAACGAGTAATTATTAATTGCCATGCCAGTGAGATCATT
AATACTTGGTTATCCTGGCACCAGCAGAAACCAGAGAATGCGCCACAACTACTCATCTCTAAGGCATCCAACTTCCATACTGAGGT
TCCATCAAGGTTCAGTGGCAGTGGATCTGGAACAGATTACTCTCTCAGCAGCAGCAGCCTCGAGCCTGAAGACATTGCTACTTACT
ACTGTGTACAGGCTAAGAGTCTTCCTCC
