# Supplementary material for: Massively parallel single-cell B-cell receptor sequencing enables rapid discovery of diverse antigen-reactive antibodies
Source: Commun Biol. 2019 Aug 9;2:304. doi: 10.1038/s42003-019-0551-y (PMC6689056; doi:10.1038/s42003-019-0551-y)
Supplement: Supplementary file 2 — Description of additional supplementary items [file 42003_2019_551_MOESM2_ESM.docx]

**DESCRIPTION OF ADDITIONAL SUPPLEMENTARY ITEMS**

**Supplementary Data 1**

Summary of cell numbers, pairing efficiencies and filtering rates by sample (a) or library (b).

**Supplementary Data 2-11**

BCR information for B-cell repertoires from rat, mouse and human samples.

**Supplementary Data 12**

BCR information for OVA antigen-reactive B cells, including VH and VL nucleotide and protein sequences.

**Supplementary Data 13**

OVA antigen-reactive B cell lineages. Read count and SHM load were obtained by averaging across all cells in the same lineage.

**Supplementary Data 14**

Source data for read coverage (Figure 2c, Supplementary Figure 5, Supplementary Figure 11c).

**Supplementary Data 15**

Source data for comparison of unique VH-VL nucleotide sequences obtained by scBCR-seq and reference VH-VL pairs obtained by a standard sequencing approach (Figure 3a).
