# Supplementary material for: Massively parallel single-cell B-cell receptor sequencing enables rapid discovery of diverse antigen-reactive antibodies
Source: Commun Biol. 2019 Aug 9;2:304. doi: 10.1038/s42003-019-0551-y (PMC6689056; doi:10.1038/s42003-019-0551-y)
Supplement: Supplementary file 4 — Reporting Summary [file 42003_2019_551_MOESM4_ESM.pdf]

natureresearch

Corresponding author(s): Leonard Goldstein, Isidro Hötzel, Somasekar Seshagiri

Last updated by author(s): July 10, 2019

# Reporting Summary

Nature Research wishes to improve the reproducibility of the work that we publish. This form provides structure for consistency and transparency in reporting. For further information on Nature Research policies, see Authors & Referees and the Editorial Policy Checklist.

## Statistics

For all statistical analyses, confirm that the following items are present in the figure legend, table legend, main text, or Methods section.

| n/a | Confirmed | |
|---|---|---|
| ☐ | ☒ | The exact sample size (*n*) for each experimental group/condition, given as a discrete number and unit of measurement |
| ☐ | ☒ | A statement on whether measurements were taken from distinct samples or whether the same sample was measured repeatedly |
| ☒ | ☐ | The statistical test(s) used AND whether they are one- or two-sided<br>*Only common tests should be described solely by name; describe more complex techniques in the Methods section.* |
| ☒ | ☐ | A description of all covariates tested |
| ☒ | ☐ | A description of any assumptions or corrections, such as tests of normality and adjustment for multiple comparisons |
| ☒ | ☐ | A full description of the statistical parameters including central tendency (e.g. means) or other basic estimates (e.g. regression coefficient) AND variation (e.g. standard deviation) or associated estimates of uncertainty (e.g. confidence intervals) |
| ☒ | ☐ | For null hypothesis testing, the test statistic (e.g. *F*, *t*, *r*) with confidence intervals, effect sizes, degrees of freedom and *P* value noted<br>*Give P values as exact values whenever suitable.* |
| ☒ | ☐ | For Bayesian analysis, information on the choice of priors and Markov chain Monte Carlo settings |
| ☒ | ☐ | For hierarchical and complex designs, identification of the appropriate level for tests and full reporting of outcomes |
| ☐ | ☒ | Estimates of effect sizes (e.g. Cohen's *d*, Pearson's *r*), indicating how they were calculated |

*Our web collection on statistics for biologists contains articles on many of the points above.*

## Software and code

Policy information about availability of computer code

| | |
|---|---|
| Data collection | Illumina bcl2fastq (2.20) |
| Data analysis | SSAKE (3.8.5), Absolve (https://github.com/Genentech/Absolve) |

For manuscripts utilizing custom algorithms or software that are central to the research but not yet described in published literature, software must be made available to editors/reviewers. We strongly encourage code deposition in a community repository (e.g. GitHub). See the Nature Research guidelines for submitting code & software for further information.

## Data

Policy information about availability of data

All manuscripts must include a data availability statement. This statement should provide the following information, where applicable:

- Accession codes, unique identifiers, or web links for publicly available datasets
- A list of figures that have associated raw data
- A description of any restrictions on data availability

Rat and mouse datasets generated during the current study are available at the NCBI Sequence Read Archive under project number PRJNA544118. Human datasets generated during the current study have been deposited at the European Genome-phenome Archive under accession code EGAS00001003663. Source data underlying Figures 2-6 are included in Supplementary Data 1-15.

# Field-specific reporting

Please select the one below that is the best fit for your research. If you are not sure, read the appropriate sections before making your selection.

☒ Life sciences ☐ Behavioural & social sciences ☐ Ecological, evolutionary & environmental sciences

For a reference copy of the document with all sections, see nature.com/documents/nr-reporting-summary-flat.pdf

# Life sciences study design

All studies must disclose on these points even when the disclosure is negative.

| | |
|---|---|
| Sample size | 2-3 samples for repertoire datasets, 1 sample for antigen-positive dataset (pooled from three animals) |
| Data exclusions | No data were excluded from the analyses |
| Replication | Reproducibility was verified using replicate samples and publicly available data sets, as indicated |
| Randomization | N/A |
| Blinding | N/A |

# Reporting for specific materials, systems and methods

We require information from authors about some types of materials, experimental systems and methods used in many studies. Here, indicate whether each material, system or method listed is relevant to your study. If you are not sure if a list item applies to your research, read the appropriate section before selecting a response.

## Materials & experimental systems

| n/a | Involved in the study | |
|---|---|---|
| ☐ | ☒ | Antibodies |
| ☐ | ☒ | Eukaryotic cell lines |
| ☒ | ☐ | Palaeontology |
| ☐ | ☒ | Animals and other organisms |
| ☐ | ☒ | Human research participants |
| ☒ | ☐ | Clinical data |

## Methods

| n/a | Involved in the study | |
|---|---|---|
| ☒ | ☐ | ChIP-seq |
| ☐ | ☒ | Flow cytometry |
| ☒ | ☐ | MRI-based neuroimaging |

## Antibodies

Antibodies used

anti-human IgG APC (Jackson Immunoresearch, cat # 109-606-170, goat polyclonal, Lot # 121188), anti-human CD20 PE-Cy7 (BD Biosciences, cat # 560735, clone 2H7, Lot # 4283782), anti-human CD4 APC-Cy7 (BD Biosciences, cat # 557871, clone RPA-74, Lot # 21580), anti-human CD56 PE (BD Biosciences, cat # 340363, clone NCAM 16.2 , Lot # 5113965), anti-human CD11c PE (BD Biosciences, cat # 340713, clone S-HCC-3, Lot # 5057818), anti-human CD8 PE (BD Biosciences, cat # 555635, clone HIT#8A, Lot # 4364551), anti-human CD11b PE (BD Biosciences, cat # 347557, clone D12, Lot # 5050767), anti-human CD14 PE (BD Biosciences, cat # 340683, clone MOP9, Lot # 5050826), anti-human CD16 PE (BD Biosciences, cat # 340705, clone B73.1, Lot # 6112553), anti-human 64 PE (BD Biosciences, cat # 644385, clone 10.1, Lot # 5182937),
Mouse B cell Enrichment Kit (Miltenyi, cat # 130-090-862, lot # 5170526618), anti-mouse B220 APC (BD Biosciences, cat # 553092, clone RA3-6B2, Lot # 4073804), anti-mouse IgG FITC (Bethyl Laboratories, cat # A90-239F, goat polyclonal), anti-rat CD4 (BD Biosciences, cat # 554836, clone OX-35, Lot # 7118728), anti-rat CD8a (BD Biosciences, cat # 554855, clone OX-8, Lot # 7194744), anti-rat CD11b/c (BD Biosciences, cat # 201803, clone OX-42, Lot # 13153115), anti-rat CD161 (BD Biosciences, cat # 550978, clone 10/78, Lot # 4050509), anti-rat granulocyte (BD Biosciences, cat # 13-0570-82, clone HIS-48, Lot # E02575-1633), anti-rat IgM PE-Cy7 (BD Biosciences, cat # 553886, clone G53-238, Lot # 3053817), anti-rat IgG Alexa488 (Jackson Immunoresearch, cat # 112-096-071, goat polyclonal, Lot # 136516), anti-rat IgG HRP (Jackson Immunoresearch, cat # 112-036-071, goat polyclonal, Lot # 136397).
Anti rat CD45RA APC Cy7 (cat# 561624, clone OX-33, lot# 5142809)
anti rat granulocyte BV510 (BD Biosciences, cat# 554905 clone HIS48, Lot# 624144)
anti rat CD11b/c BV510 (BD Biosciences, cat# 554859 clone OX42, Lot# 624144)
anti rat CD161a BV510 (BD Biosciences, cat# 555006 clone 10/78, Lot# 624144)
anti rat CD4 BV510 (BD Biosciences, cat# 554835 clone OX35, Lot# 624144), and anti rat CD8a PerCP (ThermoFisher Scientific, cat# 46-0084-82, clone OX8, Lot# E16536-101)
Concentrations of antibodies used per manufacturer's recommendations.

Validation

anti-human IgG APC (https://www.jacksonimmuno.com/catalog/products/109-606-), anti-human CD20 PE-Cy7 (http://www.bdbiosciences.com/us/applications/research/stem-cell-research/hematopoietic-stem-cell-markers/human/negative-markers/pe-cf594-mouse-anti-human-cd20-2h7/p/562322), anti-human CD4 APC-Cy7 (http://www.bdbiosciences.com/eu/

applications/research/t-cell-immunology/th-1-cells/surface-markers/human/apc-cy7-mouse-anti-human-cd4-rpa-t4/p/557871), anti-human CD56 PE (http://www.bdbiosciences.com/us/applications/research/stem-cell-research/hematopoietic-stem-cell-markers/human/negative-markers/pe-mouse-anti-human-cd56-ncam162-also-known-as-ncam-16/p/340363), anti-human CD11c PE (http://www.bdbiosciences.com/eu/reagents/clinical/reagents/single-antibodies/cd11c-pe-s-hcl-3/p/333149), anti-human CD8 PE (http://www.bdbiosciences.com/eu/reagents/research/antibodies-buffers/immunology-reagents/anti-human-antibodies/cell-surface-antigens/pe-mouse-anti-human-cd8-hit8a/p/555635), anti-human CD11b PE (http://www.bdbiosciences.com/us/applications/research/stem-cell-research/mesenchymal-stem-cell-markers-bone-marrow/human/negative-markers/pe-mouse-anti-human-cd11b-d12/p/347557), anti-human CD14 PE (https://www.bdbiosciences.com/us/applications/clinical/blood-cell-disorders/asr-reagents/cd14-pe-mp9-also-known-as-mp-9/p/340683), anti-human CD16 PE (http://www.bdbiosciences.com/us/applications/clinical/blood-cell-disorders/asr-reagents/cd16-pe-b731/p/340705), anti-human 64 PE (https://www.bdbiosciences.com/us/reagents/research/clinical-research---ruo-gmp/single-color-antibodies/pe-mouse-anti-human-cd64-101/p/644385),
Mouse B cell Enrichment Kit (https://www.miltenyibiotec.com/US-en/products/macs-cell-separation/cell-separation-reagents/microbeads-and-isolation-kits/b-cells/b-cell-isolation-kit-mouse.html), anti-mouse B220 APC (http://www.bdbiosciences.com/eu/applications/research/stem-cell-research/hematopoietic-stem-cell-markers/mouse/negative-markers/apc-rat-anti-mouse-cd45rb220-ra3-6b2/p/553092), anti-mouse IgG FITC (https://www.bethyl.com/product/A90-239F), anti-rat CD4 (http://www.bdbiosciences.com/ca/applications/research/t-cell-immunology/th-1-cells/surface-markers/rat/biotin-mouse-anti-rat-cd4-ox-35/p/554836), anti-rat CD8a (https://www.bdbiosciences.com/us/reagents/research/antibodies-buffers/immunology-reagents/anti-rat-antibodies/cell-surface-antigens/purified-mouse-anti-rat-cd8a-ox-8/p/550298), anti-rat CD11b/c (http://www.bdbiosciences.com/us/applications/research/b-cell-research/surface-markers/rat/purified-mouse-anti-rat-cd11bc-ox-42/p/554859), anti-rat CD161 (https://www.bdbiosciences.com/ca/reagents/research/antibodies-buffers/immunology-reagents/anti-rat-antibodies/cell-surface-antigens/biotin-mouse-anti-rat-cd161a-1078/p/550978), anti-rat granulocyte (http://www.bdbiosciences.com/us/applications/research/stem-cell-research/hematopoietic-stem-cell-markers/human/negative-markers/purified-mouse-anti-human-cd48-t145/p/555758), anti-rat IgM PE-Cy7 (http://www.bdbiosciences.com/eu/applications/research/b-cell-research/immunoassays/elisa/pairs-and-standards/other-species/purified-mouse-anti-rat-igm-g53-238/p/553885), anti-rat IgG Alexa488 (https://www.jacksonimmuno.com/catalog/products/112-096-071), anti-rat IgG HRP (https://www.jacksonimmuno.com/catalog/products/112-036-071).
BV510 anti rat granulocyte (http://www.bdbiosciences.com/eu/reagents/research/antibodies-buffers/immunology-reagents/anti-rat-antibodies/cell-surface-antigens/purified-mouse-anti-rat-granulocytes-his48/p/554905)
BV510 anti rat CD11b/c (http://www.bdbiosciences.com/us/applications/research/b-cell-research/surface-markers/rat/purified-mouse-anti-rat-cd11bc-ox-42/p/554859)
BV510 anti rat CD161a (http://www.bdbiosciences.com/us/reagents/research/antibodies-buffers/immunology-reagents/anti-rat-antibodies/cell-surface-antigens/bv510-mouse-anti-rat-cd161a-1078/p/744050)
BV510 anti rat CD4 (http://www.bdbiosciences.com/us/reagents/research/antibodies-buffers/immunology-reagents/anti-rat-antibodies/cell-surface-antigens/bv510-mouse-anti-rat-cd4-ox-38/p/743089)
and anti rat CD8a PerCP (https://www.thermofisher.com/antibody/product/CD8a-Antibody-clone-OX8-Monoclonal/46-0084-82)
Anti rat CD45RA APC Cy7 (http://www.bdbiosciences.com/eu/applications/research/b-cell-research/surface-markers/rat/apc-cy7-mouse-anti-rat-cd45ra-ox-33/p/561624)

# Eukaryotic cell lines

Policy information about cell lines

| | |
|---|---|
| Cell line source(s) | ThermoFisher Scientific (Expi293F) |
| Authentication | By vendor |
| Mycoplasma contamination | Negative |
| Commonly misidentified lines (See ICLAC register) | Not Applicable |

# Animals and other organisms

Policy information about studies involving animals; ARRIVE guidelines recommended for reporting animal research

| | |
|---|---|
| Laboratory animals | Mouse (Balb/c, female, 6-8 weeks), Rat (Sprague-Dawley, female, 8-12 weeks) |
| Wild animals | Not applicable |
| Field-collected samples | Not applicable |
| Ethics oversight | All animals used in this study were housed and maintained at Genentech in accordance with American Association of Laboratory Animal Care guidelines. All experimental studies were conducted under protocols approved by the Institutional Animal Care and Use Committee of Genentech Lab Animal Research in an Association for Assessment and Accreditation of Laboratory Animal Care International-accredited facility in accordance with the Guide for the Care and Use of Laboratory Animals and applicable laws and regulations. |

Note that full information on the approval of the study protocol must also be provided in the manuscript.

## Human research participants

Policy information about studies involving human research participants

| | |
|---|---|
| Population characteristics | Blood donor information is blinded |
| Recruitment | Volunteers for Genentech Research Blood Program |
| Ethics oversight | Human blood from healthy human donors was obtained after written informed consent was provided and ethical approval was granted by the Western Institutional Review Board. |

Note that full information on the approval of the study protocol must also be provided in the manuscript.

## Flow Cytometry

### Plots

Confirm that:

☒ The axis labels state the marker and fluorochrome used (e.g. CD4-FITC).

☒ The axis scales are clearly visible. Include numbers along axes only for bottom left plot of group (a 'group' is an analysis of identical markers).

☒ All plots are contour plots with outliers or pseudocolor plots.

☒ A numerical value for number of cells or percentage (with statistics) is provided.

### Methodology

| | |
|---|---|
| Sample preparation | Detailed in the manuscript |
| Instrument | Detailed in the manuscript |
| Software | BD FACSDiva software |
| Cell population abundance | Purity of sorted cell population in post-sort cells was >95% as determined by analyses of post-sort cells in flow cytometer after sorting. |

Gating strategy

Human IgG+ B cell sort gating strategy
1) FSC vs SSC to gate lymphocytes
2/3) SSC-H/SSC-W, FSC-H/FSC-W gates to exclude cell doublets
4) FSC/PI for dead cell exclusion (PI-negative gate)
5) IgG-APC/Dump-PE (CD11b, CD11c, CD14, CD16, CD56, CD64, CD8) to exclude PE-positive non-B cells
6) CD20-PE Cy7/CD4 APC-Cy7 to exclude CD4+ cells
7) CD20-PECy7/IgG-APC to sort for IgG+ B cells

Naïve Balb/c mouse B cell sort gating strategy
1) FSC vs SSC to gate lymphocytes
2/3) SSC-H/SSC-W, FSC-H/FSC-W gates to exclude cell doublets
4) FSC/PI for dead cell exclusion (PI-negative gate)
5) IgG FITC/B220-APC to sort for IgG+ B cells

Naïve SD rat B cell sort gating strategy
1) FSC vs SSC to gate lymphocytes
2/3) SSC-H/SSC-W, FSC-H/FSC-W gates to exclude cell doublets
4) FSC/PI for dead cell exclusion (PI-negative gate)
5) CD8-PerCP Cy5.5/Dump-BV510 (CD4, CD11b, CD161a, granulocyte marker) to exclude CD8+BV510+ non-B cells
6) IgM-PECy7/CD45RA-APC Cy7 to sort for B cells

Immunized SD rat, OVA+IgM- B cell sort gating strategy
1) FSC vs SSC to gate lymphocytes
2/3) SSC-H/SSC-W, FSC-H/FSC-W to to exclude cell doublets
4) FSC/PI for dead cell exclusion (PI-negative gate)
5) CD8-PerCP Cy5.5/Dump-BV510 (CD4, CD11b, CD161a, granulocyte marker) to exclude BV510+ non-B cells
6) CD45R-APC Cy7/CD8-PerCP Cy5.5 to exclude CD8+ cells
7) IgM-PE Cy7/CD45R-APC Cy7 to gate for IgM- B cells
8) CD45R-APC Cy7/OVA-APC to sort for OVA+IgM- B cells

Immunized SD rat, OVA+IgG+ hybridoma sort gating strategy
1) FSC vs SSC to gate hybridoma cells
2/3) SSC-H/SSC-W, FSC-H/FSC-W gates to exclude cell doublets
4) FSC/PI for dead cell exclusion (PI-negative gate)

5) IgG FITC/BV421 (empty channel) to gate for IgG+ hybridomas
6) PE (empty channel)/OVA-APC to sort for OVA+IgG+ hybridomas

☒ Tick this box to confirm that a figure exemplifying the gating strategy is provided in the Supplementary Information.
